# An efficient exact method to obtain GBLUP and single-step GBLUP when the genomic relationship matrix is singular

**DOI:** 10.1186/s12711-016-0260-7

**Published:** 2016-10-27

**Authors:** Rohan L. Fernando, Hao Cheng, Dorian J. Garrick

**Affiliations:** 1 Department of Animal Science, Iowa State University, Ames, IA 50011 USA; 2 Institute of Veterinary, Animal and Biomedical Sciences, Massey University, Palmerston North, New Zealand

## Abstract

**Background:**

The mixed linear model employed for genomic best linear unbiased prediction (GBLUP) includes the breeding value for each animal as a random effect that has a mean of zero and a covariance matrix proportional to the genomic relationship matrix (${\mathbf {G}}_{gg}$), where the inverse of ${\mathbf {G}}_{gg}$ is required to set up the usual mixed model equations (MME). When only some animals have genomic information, genomic predictions can be obtained by an extension known as single-step GBLUP, where the covariance matrix of breeding values is constructed by combining the pedigree-based additive relationship matrix with ${\mathbf {G}}_{gg}$. The inverse of the combined relationship matrix can be obtained efficiently, provided ${\mathbf {G}}_{gg}$ can be inverted. In some livestock species, however, the number $N_{g}$ of animals with genomic information exceeds the number of marker covariates used to compute ${\mathbf {G}}_{gg}$, and this results in a singular ${\mathbf {G}}_{gg}$. For such a case, an efficient and exact method to obtain GBLUP and single-step GBLUP is presented here.

**Results:**

Exact methods are already available to obtain GBLUP when ${\mathbf {G}}_{gg}$ is singular, but these require working with large dense matrices. Another approach is to modify ${\mathbf {G}}_{gg}$ to make it nonsingular by adding a small value to all its diagonals or regressing it towards the pedigree-based relationship matrix. This, however, results in the inverse of ${\mathbf {G}}_{gg}$ being dense and difficult to compute as $N_{g}$ grows. The approach presented here recognizes that the number *r* of linearly independent genomic breeding values cannot exceed the number of marker covariates, and the mixed linear model used here for genomic prediction only fits these *r* linearly independent breeding values as random effects.

**Conclusions:**

The exact method presented here was compared to Apy-GBLUP and to Apy single-step GBLUP, both of which are approximate methods that use a modified ${\mathbf {G}}_{gg}$ that has a sparse inverse which can be computed efficiently. In a small numerical example, predictions from the exact approach and Apy were almost identical, but the MME from Apy had a condition number about 1000 times larger than that from the exact approach, indicating ill-conditioning of the MME from Apy. The practical application of exact SSGBLUP is not more difficult than implementation of Apy.

**Electronic supplementary material:**

The online version of this article (doi:10.1186/s12711-016-0260-7) contains supplementary material, which is available to authorized users.

## Background

In animal breeding, two equivalent mixed linear models have been used for genomic prediction using phenotypes on genotyped individuals [1]. In the first, random effects of markers are explicitly included in the model [2, 3]. We will refer to this model as the marker effects model (MEM). In the second, the breeding value of each animal, which is a linear combination of the random marker effects, is included as a random effect [1, 2, 4, 5]. We will refer to this model as the breeding value model (BVM). The mixed model equations (MME) that corresponds to the MEM has order $p+k$, where *p* is the number of non-genetic effects and *k* is the number of marker covariates, and the MME that correspond to the BVM has order $p+N_{g}$, where $N_{g}$ is the number of animals. When genomic data were first available, the number $N_{g}$ of animals with genotypic and phenotypic records was much smaller than the number *k* of marker effects. Thus, genomic prediction with the BVM was more efficient than using the MEM [1, 5], and prediction using this approach is now known as GBLUP.

However, at present, in some livestock species such as dairy cattle, $N_{g}$ has increased to over 100,000 if not 1 million. When $N_{g}$ exceeds *k*, the matrix ${\mathbf {G}}_{gg}$ of genomic relationships will have at least $n-k$ eigen values that are zero, and therefore ${\mathbf {G}}_{gg}$ is guaranteed to be singular. In practice, depending on the effective population size, some of the smallest of the *k* largest eigen values may be very near to zero if not zero. In either event, the MME that require the inverse of ${\mathbf {G}}_{gg}$ cannot be employed to obtain GBLUP. In that situation, an alternate form of the MME [4, 6–8] that can accommodate a singular ${\mathbf {G}}_{gg}$ can be employed, but this results in a completely dense set of MME of order $p+N_{g}$. Thus, when $N_{g}$ is large, this formulation of the MME is not useful for computing GBLUP. An alternative is to use a modified matrix ${\mathbf {G}}^{*}$ obtained from ${\mathbf {G}}_{gg}$ by adding a small value to all its diagonals or by regressing it towards the pedigree-based relationship matrix, ${\mathbf {A}}$, so that it retains full rank, but this is no longer an exact representation of the model if the markers completely explain the breeding values. Furthermore, this modified relationship matrix still has a dense inverse, which may be impossible to compute when $N_{g}$ is large.

Suppose the rank of ${\mathbf {G}}_{gg}$ is $r\le k<N_{g}$. Then, we will show here how to obtain exact GBLUP without approximation from a set of MME that has order $p+r$, which can be much lower than $p+N_{g}.$ We also show how this approach can be used to obtain exact single-step GBLUP without approximation when some animals have not been genotyped. These formulations are useful to better understand predictions that are obtained by using the recursive algorithm for “parents (core)” and “young (noncore)” animals i.e. Apy, which is gaining popularity [9–13] as an approach to approximate the inverse of ${\mathbf {G}}_{gg}$ [9] or ${\mathbf {G}}^{*}$ [13]. The exact inverse of the nonsingular matrix ${\mathbf {G}}^{*}=0.95{\mathbf {G}}_{gg}+0.05{\mathbf {A}}$ will be dense whereas Apy approximates this with a sparse inverse [9, 10]. We will show here that when a full-rank ${\mathbf {G}}^{*}$ is obtained by adding a small number to the diagonals of only noncore animals, the inverse calculated in Apy for a suitable choice of core animals will be sparse and an exact inverse of ${\mathbf {G}}^{*}$, but the inverse may be ill conditioned. The approximate inverse calculated in Apy cannot ever be that of ${\mathbf {G}}_{gg},$ which is singular when $r<N_{g}$. The Apy algorithm will never yield exact GBLUP predictions contrary to the claims in [9, 11], but it has been demonstrated to be a useful approximation for some choices of ${\mathbf {G}}^{*}$ [11–13].

## Theory

Let ${\mathbf {M}}_{g}$ denote the centered marker genotype covariate matrix of order $N_{g}\times k$ with $N_{g}>k,$ which is the case when the number $N_{g}$ of genotyped animals is larger than the number *k* of marker covariates. Then, the row rank *r* of ${\mathbf {M}}_{g}$ is $r\le k<N_{g}$ [14]. Suppose ${\mathbf {M}}_{g}$ is ordered such that its first *r* rows are linearly independent and are denoted ${\mathbf {M}}_{g_{i}}$. It follows that the remaining $N_{g}-r$ dependent rows of ${\mathbf {M}}_{g}$, denoted ${\mathbf {M}}_{g_{d}}$ can be written as a linear combination:

$$\begin{aligned} {\mathbf {M}}_{g_{d}}={\mathbf {L}}^{\prime}{\mathbf {M}}_{g_{i}}, \end{aligned}$$

so that

1 $$\begin{aligned} {\mathbf {M}}_{g} &= \begin{bmatrix}{\mathbf {M}}_{g_{i}}\\ {\mathbf {M}}_{g_{d}} \end{bmatrix}\nonumber \\ &= \begin{bmatrix}{\mathbf {M}}_{g_{i}}\\ {\mathbf {L}}^{\prime}{\mathbf {M}}_{g_{i}} \end{bmatrix}. \end{aligned}$$

Now, a commonly-used form of the genomic relationship matrix [5] becomes

2 $$\begin{aligned} {\mathbf {G}}_{gg} &= \frac{{\mathbf {M}}_{g}{\mathbf {M}}_{g}^{\prime}}{k}\nonumber \\ & = \frac{1}{k}\left[ \begin{array}{cc} {\mathbf {M}}_{g_{i}}{\mathbf {M}}^{\prime}_{g_{i}} & \quad {\mathbf {M}}_{g_{i}}{\mathbf {M}}^{\prime}_{g_{i}}{\mathbf {L}}\\ {\mathbf {L}}^{\prime}{\mathbf {M}}_{g_{i}}{\mathbf {M}}^{\prime}_{g_{i}} & \quad {\mathbf {L}}^{\prime}{\mathbf {M}}_{g_{i}}{\mathbf {M}}^{\prime}_{g_{i}}{\mathbf {L}} \end{array}\right] , \end{aligned}$$

where it can be seen that the last $N_{g}-r$ rows are a linear combination of the first *r* rows. The last $N_{g}-r$ columns of ${\mathbf {G}}$ are similarly a linear combination of the first *r* columns. Thus, in this case, ${\mathbf {G}}$ is singular and its inverse does not exist. It can be seen from (2) that ${\mathbf {L}}^{\prime}$ can be written as:

3 $$\begin{aligned} {\mathbf {L}}^{\prime}={\mathbf {G}}_{g_{d}g_{i}}{\mathbf {G}}_{g_{i}g_{i}}^{-1}, \end{aligned}$$

where $${\mathbf {G}}_{g_{d}g_{i}}=\frac{1}{k}{\mathbf {M}}_{g_{d}}{\mathbf {M}}^{\prime}_{g_{i}}$$ and $${\mathbf {G}}_{g_{i}g_{i}}=\frac{1}{k}{\mathbf {M}}_{g_{i}}{\mathbf {M}}^{\prime}_{g_{i}}.$$

### GBLUP when G is singular

In the following, we will assume that the vector ${\mathbf {u}}_{g}$ of breeding values of animals can be adequately modeled as:

4 $$\begin{aligned} {\mathbf {u}}_{g}={\mathbf {M}}_{g}\varvec{\alpha }, \end{aligned}$$

where the vector $\varvec{\alpha }$ of marker effects is assumed to have zero mean and covariance matrix ${\mathbf {I}}\sigma _{\alpha }^{2}.$ It follows that the covariance matrix of the breeding values is:

5 $$\begin{aligned} \text {Var}({\mathbf {u}}_{g}|{\mathbf {M}}_{g})&= {\mathbf {M}}_{g}{\mathbf {M}}_{g}^{\prime}\sigma _{\alpha }^{2} \\ & = {\mathbf {G}}_{gg}k\sigma _{\alpha }^{2}\\ & = {\mathbf {G}}_{gg}\sigma _{u}^{2}, \end{aligned}$$

where $\sigma _{u}^{2}=k\sigma _{\alpha }^{2}.$ To proceed, we further assume the following mixed linear model for the vector ${\mathbf {y}}$ of phenotypic values:

6 $$\begin{aligned} {\mathbf {y}}={\mathbf {X}}\varvec{\beta }+{{\mathbf {Z}}}{{\mathbf {u}}}_{g}+{\mathbf {e}}, \end{aligned}$$

where $\varvec{\beta }$ is a vector of non-genetic fixed effects, ${\mathbf {X}}$ and ${\mathbf {Z}}$ are incidence matrices relating $\varvec{\beta }$ and ${\mathbf {u}}_{g}$ to ${\mathbf {y}},$ and ${\mathbf {e}}$ is a vector of residuals with zero mean and covariance matrix ${\mathbf {I}}\sigma _{e}^{2}.$ Here, we have assumed that the markers fully explain the breeding values. If this is not the case, a random polygenic residual effect with zero mean and covariance matrix that is proportional to ${\mathbf {A}}$ can be included in the model.

#### Strategy I

When ${\mathbf {G}}$ is singular, one strategy to get the BLUP of ${\mathbf {u}}$ is to use the formula:

7 $$\begin{aligned} \hat{{\mathbf {u}}}_{g} &= \text {Cov}({\mathbf {u}}_{g},{\mathbf {y}}^{\prime})\text {Var}^{-1}({\mathbf {y}})({\mathbf {y}}-{\mathbf {X}}\hat{\varvec{\beta }}) \\ &= {\mathbf {G}}_{gg}{\mathbf {Z}}^{\prime}{\mathbf {V}}^{-1}({\mathbf {y}}-{\mathbf {X}}\hat{\varvec{\beta }}), \end{aligned}$$

where $\hat{\varvec{\beta }}$ is a solution to the system

8 $$\begin{aligned} {(}{\mathbf {X}}^{\prime}{\mathbf {V}}^{-1}{\mathbf {X}}{)}\hat{\varvec{\beta }}={\mathbf {X}}^{\prime}{\mathbf {V}}^{-1}{\mathbf {y}}, \end{aligned}$$

and ${\mathbf {V}}=({{\mathbf {Z}}}{{\mathbf {G}}}_{gg}{\mathbf {Z}}^{\prime}\sigma _{u}^{2}+{\mathbf {I}}\sigma _{e}^{2})$ [7]. When $N_{g}$ is large, this strategy is not computationally feasible because the matrix ${\mathbf {V}}$ is dense, has order $N_{g}$, and its inverse is needed in (7) and (8).

#### Strategy II

Another strategy is to get the solution to the following MME as proposed by Harville [6]:

9 $$\begin{aligned} \left[ \begin{array}{cc} {\mathbf {X}}^{\prime}{\mathbf {R}}^{-1}{\mathbf {X}} & \quad {\mathbf {X}}^{\prime}{\mathbf {R}}^{-1}{\mathbf {Z}}\\ {\mathbf {G}}_{gg}{\mathbf {Z}}^{\prime}{\mathbf {R}}^{-1}{\mathbf {X}} & \quad {\mathbf {G}}_{gg}{\mathbf {Z}}^{\prime}{\mathbf {R}}^{-1}{\mathbf {Z}}+{\mathbf {I}} \end{array}\right] \left[ \begin{array}{c} \hat{\varvec{\beta }}\\ \hat{{\mathbf {u}}}_{g} \end{array}\right] =\left[ \begin{array}{c} {\mathbf {X}}^{\prime}{\mathbf {R}}^{-1}{\mathbf {y}}\\ {\mathbf {G}}_{gg}{\mathbf {Z}}^{\prime}{\mathbf {R}}^{-1}{\mathbf {y}} \end{array}\right] , \end{aligned}$$

where $\mathbf {R^{-1}}={\mathbf {I}}\frac{1}{\sigma _{e}^{2}}.$ These MME are dense and have order $p+N_{g}$. Thus, although the above approaches do not require inverting ${\mathbf {G}}_{gg}$, explicitly using these MME do not provide a feasible approach as the number $N_{g}$ of genotyped animals approaches or exceeds a million, because storing and solving such large and dense system of equations would exceed the capacity of the typical computer used for genetic evaluation. An implementation with iteration on data using the PCG algorithm may be feasible by computing matrix products like ${\mathbf {G}}_{gg}{\mathbf {x}}$ in parts as $\frac{1}{k}\mathbf {M_g}(\mathbf {M_g}^{\prime}{\mathbf {x}})$ [15]. However, Aguilar et al. [16] reported these asymmetric equations do not scale up well and suffer convergence problems.

#### Strategy III

We show here that it is possible to obtain BLUP of ${\mathbf {u}}_{g}$ by solving a set of MME that has order $p+r$, which can be much lower than $p+N_{g}$. To do so, the breeding values of the *r* animals with genotypes ${\mathbf {M}}_{g_{i}}$ is denoted ${\mathbf {u}}_{g_{i}}$ and the breeding values of the $N_{g}-r$ animals with genotypes ${\mathbf {M}}_{g_{d}}$ is denoted ${\mathbf {u}}_{g_{d}}.$ The model for the breeding values in (4) can be written as:

10 $$\begin{aligned} {\mathbf {u}}_{g} & = {\mathbf {M}}_{g}\varvec{\alpha }\\ &= \begin{bmatrix}{\mathbf {M}}{}_{g_{i}}\\ {\mathbf {M}}{}_{g_{d}} \end{bmatrix}\varvec{\alpha },\nonumber \end{aligned}$$

and writing ${\mathbf {M}}_{g_{d}}={\mathbf {L}}^{\prime}{\mathbf {M}}_{g_{i}}$ as in (1), this becomes:

11 $$\begin{aligned} {\mathbf {u}}_{g} & = \begin{bmatrix}{\mathbf {M}}_{g_{i}}\\ {\mathbf {L}}^{\prime}{\mathbf {M}}{}_{g_{i}} \end{bmatrix}\varvec{\alpha }\nonumber \\ &= \begin{bmatrix}{\mathbf {I}}\\ {\mathbf {L}}^{\prime} \end{bmatrix}{\mathbf {M}}{}_{g_{i}}\alpha \nonumber \\ &= \begin{bmatrix}{\mathbf {I}}\\ {\mathbf {L}}^{\prime} \end{bmatrix}{\mathbf {u}}_{g_{i}}. \end{aligned}$$

Note that the vector of breeding values given by (11) is identical to (4), and thus these two vectors have the same covariance matrix that is given by (5).

Now, using (11) for ${\mathbf {u}}_{g}$ in (6), the mixed linear model for the phenotypic values can be written in terms of ${\mathbf {u}}_{g_{i}}$ as:

12 $$\begin{aligned} {\mathbf {y}}={\mathbf {X}}\varvec{\beta }+{\mathbf {Z}} \begin{bmatrix}{\mathbf {I}}\\ {\mathbf {L}}^{\prime} \end{bmatrix}{\mathbf {u}}_{g_{i}}+{\mathbf {e}}. \end{aligned}$$

The random effect ${\mathbf {u}}_{g_{i}}$ of this model has order *r* and can be much lower than $N_{g}$ the order of ${\mathbf {u}}_{g}.$ Furthermore, as $${\mathbf {u}}_{g}=\begin{bmatrix}{\mathbf {I}}\\ {\mathbf {L}}^{\prime} \end{bmatrix}{\mathbf {u}}_{g_{i}},$$ the models given by (6) and (12) have the same first and second moments, and thus they are equivalent models and yield the same BLUP for ${\mathbf {u}}_{g}$ [7]. The MME for the model (12) are

13 $$\begin{aligned} \left[ \begin{array}{cc} {\mathbf {X}}^{\prime}{\mathbf {X}}& \quad {\mathbf {X}}^{\prime}{\mathbf {W}}\\ {\mathbf {W}}^{\prime}{\mathbf {X}}& \quad {\mathbf {W}}^{\prime}{\mathbf {W}}+\lambda {\mathbf {G}}_{g_{i}g_{i}}^{-1} \end{array}\right] \left[ \begin{array}{c} \hat{\varvec{\beta }}\\ \hat{{\mathbf {u}}}_{g_{i}} \end{array}\right] =\left[ \begin{array}{c} {\mathbf {X}}^{\prime}{\mathbf {y}}\\ {\mathbf {W}}^{\prime}{\mathbf {y}} \end{array}\right] , \end{aligned}$$

where $${\mathbf {W}}={\mathbf {Z}}\begin{bmatrix}{\mathbf {I}} \\ {\mathbf {L}}^{\prime} \end{bmatrix}, {\mathbf {G}}_{g_{i}g_{i}}^{-1}$$ is the inverse of the $r\times r$ non-singular matrix ${\mathbf {G}}_{g_{i}g_{i}}=\frac{1}{k} ({\mathbf {M}}_{g_{i}}{\mathbf {M}}^{\prime}_{g_{i}})$, and $\lambda =\frac{\sigma _{e}^{2}}{\sigma _{u}^{2}}.$ The BLUP of ${\mathbf {u}}_{g_{d}}$ is obtained as ${\hat{\mathbf{u}}}_{g_{d}}={\mathbf {L}}^{\prime}\hat{{\mathbf {u}}}_{g_{i}}.$

#### Strategy IV

A key assumption in Strategy III is that the matrix ${\mathbf {M}}_{g}$ of marker covariates can be reordered such that the first *r* rows are linearly independent and the remaining dependent rows can be expressed as a linear combination of the first set of *r* linearly independent rows. Determining the precise rank of ${\mathbf {M}}_{g}$ may be inexact as the eigen values of ${\mathbf {G}}_{gg}$ decay slowly [17]. On the one hand, if the chosen ${\mathbf {M}}_{g_{i}}$ contains less rows than the rank of ${\mathbf {G}}_{gg}$, it would not be possible to express ${\mathbf {M}}_{g_{d}}$ as ${\mathbf {M}}_{g_{d}}={\mathbf {L}}^{\prime}{\mathbf {M}}_{g_{i}}$. On the other hand, if ${\mathbf {M}}_{g_{d}}$ contains more rows than the rank of ${\mathbf {G}}_{gg}, {\mathbf {G}}_{g_{i}g_{i}}$ will be singular. Even when the number of rows in ${\mathbf {M}}_{g_{i}}$ is equal to the rank of ${\mathbf {G}}_{gg},{\mathbf {G}}_{g_{i}g_{i}}$ may be ill conditioned if the smallest eigen value of ${\mathbf {G}}_{g_ig_i}$ is close to zero. The condition number of a matrix is represented by the ratio of the largest to the smallest eigen value, and it is 1 for a perfectly conditioned matrix and a large number for an ill-conditioned matrix. There are many combinations of individuals that can be placed in ${\mathbf {M}}_{g_{i}}$, but the condition number of the resultant ${\mathbf {G}}_{g_{i}g_{i}}$ may vary greatly according to the chosen combination. The condition number of ${\mathbf {G}}_{g_{i}g_{i}}$ will impact the condition number of the resultant MME, and poorly-conditioned equations take longer to solve iteratively than well-conditioned equations. In comparing the choice of core used in Apy in a pig evaluation, Ostersen et al. [18] reported similar numbers of PCG iterations for non-genomic analyses and 8 choices of core, but the correlation between the Apy-SSGBLUP and SSGBLUP ranged from 0.93 to more than 0.99 for genotyped animals. That paper did not report the criterion used to determine PCG convergence.

One way to improve the condition number of the MME is to fit an equivalent model obtained by orthonormalizing the rows of ${\mathbf {M}}_{g_{i}}$. Suppose ${\mathbf {U}}={\mathbf {T}}{\mathbf {M}}_{g_{i}}$, where ${{\mathbf {U}}}{{\mathbf {U}}}^{\prime}={\mathbf {I}}$. Then, the transformed vector ${\mathbf {v}}={\mathbf {T}}{\mathbf {u}}_{gi}$ of breeding values will have a genomic covariance matrix:

$$\begin{aligned} \mathrm {Var}({\mathbf {v}}) &= {\mathbf {T}}\text {Var}({\mathbf {u}}_{g_{i}}){\mathbf {T}}^{\prime}\sigma _{\alpha }^{2}\\ &= {{\mathbf {T}}}{{\mathbf {M}}}_{g_{i}}{\mathbf {M}}^{\prime}_{g_{i}}{\mathbf {T}}^{\prime}\sigma _{\alpha }^{2}\\ &= {{\mathbf {U}}}{{\mathbf {U}}}^{\prime}\sigma _{\alpha }^{2}\\ &= {\mathbf {I}}\sigma _{\alpha }^{2}. \end{aligned}$$

Then, as in [17], formulating the model in terms of ${\mathbf {v}}$, which has a well-conditioned covariance matrix, will result in a well-conditioned MME.

Another way to improve the condition of the MME without explicitly reordering ${\mathbf {M}}_{g}$ is by using an RQ decomposition [19] that involves expressing ${\mathbf {M}}_{g}$ as ${\mathbf {M}}_{g}={{\mathbf {R}}}{{\mathbf {U}}},$ where ${\mathbf {R}}$ is a lower triangular $N_{g}\times k$ matrix and ${\mathbf {U}}$ is a $k\times k$ orthogonal matrix. The RQ decomposition applies to the rows of a matrix in the same manner that the QR decomposition is applied to the columns. Exploiting the decomposition, the model equation for the phenotypic values can be written in terms of ${\mathbf {v}}={\mathbf {U}}\varvec{\alpha }$ as:

$$\begin{aligned} {\mathbf {y}} &= {\mathbf {X}}\varvec{\beta }+{\mathbf {Z}}{\mathbf {M}}_{g}\varvec{\alpha }+{\mathbf {e}}\\ &= {\mathbf {X}}\varvec{\beta }+{\mathbf {Z}}{{\mathbf {R}}}{{\mathbf {U}}}\varvec{\alpha }+{\mathbf {e}}\\ &= {\mathbf {X}}\varvec{\beta }+{\mathbf {Z}}{{\mathbf {R}}}{{\mathbf {v}}}+{\mathbf {e}} \\ & = {\mathbf {X}}\varvec{\beta }+{\mathbf {W}}{\mathbf {v}}+{\mathbf {e}}, \end{aligned}$$

where now ${\mathbf {W}}={{\mathbf {Z}}}{{\mathbf {R}}}.$ The MME for this model are:

14 $$\begin{aligned} \left[ \begin{array}{cc} {\mathbf {X}}^{\prime}{\mathbf {X}} &\quad {\mathbf {X}}^{\prime}{\mathbf {W}}\\ {\mathbf {W}}^{\prime}{\mathbf {X}} & \quad {\mathbf {W}}^{\prime}{\mathbf {W}}+{\mathbf {I}}\frac{\sigma _{e}^{2}}{\sigma _{\alpha }^{2}} \end{array}\right] \left[ \begin{array}{c} \hat{\varvec{\beta }}\\ \hat{{\mathbf {v}}} \end{array}\right] =\left[ \begin{array}{c} {\mathbf {X}}^{\prime}{\mathbf {y}}\\ {\mathbf {W}}^{\prime}{\mathbf {y}} \end{array}\right] , \end{aligned}$$

and predictions for all individuals on the original scale can be obtained as $\hat{{\mathbf {u}}}_{g}={\mathbf {R}}\hat{{\mathbf {v}}}$. Also, the marker effects can be obtained as $\hat{\varvec{\alpha }}={\mathbf{U}}^{\prime} {\hat{{\mathbf{v}}}}$. Note that this factorization does not require us to know or determine the rank of ${\mathbf {M}}_{g}$. Furthermore, the orthogonal matrix ${\mathbf {U}}$ can be obtained by applying the RQ factorization to just the first *k* rows of ${\mathbf {M}}_g$, for which the number of operations is proportional to $k^3$ [19]. The matrix ${\mathbf {R}}$ can be obtained as ${\mathbf {R}} = {\mathbf {M}}_g{\mathbf {U}}^{\prime}$.

#### Comparison to Apy-GBLUP

The efficient algorithm to obtain the inverse of the additive relationship matrix is based on the property that the additive relationships between an animal and any non-descendant (an individual that is not a descendant) can be written as a linear combination of the relationships between the non-descendant and the parents of the animal [20, 21]. This property of additive relationships also allows construction of the additive relationship matrix by the tabular method [22]. The so-called Apy algorithm [9, 10] attempts to extend this idea to genomic relationships by classifying animals into two groups: “core” and “noncore” animals. The Apy algorithm seems to imply that the relationship between a noncore animal and any other animal can be written as a linear combination of relationships between the other animal and the animals in the core group. We will refer to this property of the genomic relationships that is required for Apy as the Apy property. Provided this property holds, it is claimed that Apy results in an efficient inverse of ${\mathbf {G}}_{gg}$ that leads to exact calculations of GBLUP [9, 11]. However, when $N_{g}>k, {\mathbf {G}}_{gg}$ is singular and cannot have an inverse. Thus, Apy-GBLUP cannot be exact.

To better understand the matrix portrayed as an inverse by the Apy algorithm, the genomic relationship matrix is partitioned into the core and noncore animals as follows:

$$\begin{aligned} {\mathbf {G}}_{gg}=\left[ \begin{array}{cc} {\mathbf {G}}_{cc} & \quad {\mathbf {G}}_{cn}\\ {\mathbf {G}}_{nc} & \quad {\mathbf {G}}_{nn} \end{array}\right] , \end{aligned}$$

where the subscripts *c* and *n* denote the core and noncore animals. The Apy algorithm implies that ${\mathbf {G}}_{cc}$ is nonsingular and that ${\mathbf {G}}_{nc}$ can be written as ${\mathbf {G}}_{nc}={{\mathbf {P}}}{{\mathbf {G}}}_{cc},$ where ${\mathbf {P}}={\mathbf {G}}_{nc}{\mathbf {G}}_{cc}^{-1}$. Similarly, ${\mathbf {G}}_{cn}={\mathbf {G}}_{cc}{\mathbf {P}}^{\prime}$. Now, using these results, ${\mathbf {G}}_{gg}$ can be written as:

$$\begin{aligned} {\mathbf {G}}_{gg}=\left[ \begin{array}{cc} {\mathbf {G}}_{cc} & \quad {\mathbf {G}}_{cc}{\mathbf {P}}^{\prime} \\ {{\mathbf {P}}}{{\mathbf {G}}}_{cc} & \quad {{\mathbf {P}}}{{\mathbf {G}}}_{cc}\text{P}^{\prime}+{\mathbf {D}} \end{array}\right] , \end{aligned}$$

where

15 $$\begin{aligned} {\mathbf {D}}={\mathbf {G}}_{nn}-{{\mathbf {P}}}{{\mathbf {G}}}_{cc}\text{ P }^{\prime}. \end{aligned}$$

Assuming ${\mathbf {D}}$ is nonsingular, the inverse of ${\mathbf {G}}_{gg}$ can be obtained as follows. We start by expressing ${\mathbf {G}}_{gg}$ as:

$$\begin{aligned} {\mathbf {G}}_{gg}=\left[ \begin{array}{cc} {\mathbf {I}} &{} {\mathbf {0}}\\ {\mathbf {P}} &{} {\mathbf {I}} \end{array}\right] \left[ \begin{array}{cc} {\mathbf {G}}_{cc} &{} {\mathbf {0}}\\ {\mathbf {0}} &{} {\mathbf {D}} \end{array}\right] \left[ \begin{array}{cc} {\mathbf {I}} &{} {\mathbf {P}}^{\prime}\\ {\mathbf {0}} &{} {\mathbf {I}} \end{array}\right] . \end{aligned}$$

Then, the inverse of ${\mathbf {G}}_{gg}$ can be written as:

16 $$\begin{aligned} {\mathbf {G}}_{gg}^{-1} &= \left[ \begin{array}{cc} {\mathbf {I}} &{} -{\mathbf {P}}^{\prime} \\ {\mathbf {0}} &{} {\mathbf {I}} \end{array}\right] \left[ \begin{array}{cc} {\mathbf {G}}_{cc}^{-1} &{} {\mathbf {0}}\\ {\mathbf {0}} &{} {\mathbf {D}}^{-1} \end{array}\right] \left[ \begin{array}{cc} {\mathbf {I}} &{} {\mathbf {0}}\\ -{\mathbf {P}} &{} {\mathbf {I}} \end{array}\right] \nonumber \\ &= \left[ \begin{array}{cc} {\mathbf {G}}_{cc}^{-1} &{} {\mathbf {0}}\\ {\mathbf {0}} &{} {\mathbf {0}} \end{array}\right] +\left[ \begin{array}{c} -\mathbf {P^{\prime}}\\ {\mathbf {I}} \end{array}\right] {\mathbf {D}}^{-1}\left[ \begin{array}{cc} -{\mathbf {P}}&{\mathbf {I}} \end{array}\right] , \end{aligned}$$

which is identical to the formula given in Misztal et al. [9] provided ${\mathbf {D}}$ is diagonal.

To examine the situation when $N_{g}>k$ and ${\mathbf {G}}_{gg}$ is singular, suppose the animals with genotypes in ${\mathbf {M}}_{g_{i}}$ are considered as the core animals and those with genotypes in ${\mathbf {M}}_{g_{d}}$ are considered as the noncore animals. Then, ${\mathbf {G}}_{cc}={\mathbf {G}}_{g_{i}g_{i}}, {\mathbf {G}}_{nc}={\mathbf {G}}_{g_{d}g_{i}}=\frac{1}{k}{\mathbf {L}}^{\prime}{\mathbf {M}}_{g_{i}}{\mathbf {M}}^{\prime}_{g_{i}}$, and ${\mathbf {G}}_{nn}={\mathbf {G}}_{g_{d}g_{d}}=\frac{1}{k}{\mathbf {L}}^{\prime}{\mathbf {M}}_{g_{i}}{\mathbf {M}}^{\prime}_{g_{i}}{\mathbf {L}}$, and ${\mathbf {P}}={\mathbf {L}}^{\prime},$ where from (2) ${\mathbf {L}}^{\prime}$ can also be written as ${\mathbf {L}}^{\prime}={\mathbf {G}}_{g_{d}g_{i}}{\mathbf {G}}_{g_{i}g_{i}}^{-1}$. Given this definition of the core and noncore animals, it can be seen from (2) that the Apy property holds, because the rows of ${\mathbf {G}}_{gg}$ for the noncore animals is a linear function of those in the core. Furthermore, it can be seen from (2) that:

$$\begin{aligned} {\mathbf {G}}_{nn} &= {\mathbf {G}}_{g_{d}g_{d}}\\ &= \frac{1}{k}{\mathbf {L}}{\mathbf {L}}^{\prime}{\mathbf {M}}_{g_{i}}{\mathbf {M}}^{\prime}_{g_{i}} \\ &= {\mathbf {P}}{\mathbf {G}}_{cc}{\mathbf {P}}^{\prime}, \end{aligned}$$

which shows that ${\mathbf {D}}$ is null, and thus (16) cannot be computed. In this situation, ${\mathbf {D}}$ can be replaced by ${\mathbf {I}}s$ for a positive scalar *s*. Then, (16) gives the exact inverse for a matrix ${\mathbf {G}}_{gg}^{*}$ of modified genomic relationships that is obtained by adding *s* to only the diagonals of the noncore group. If the scalar *s* is chosen to be small, ${\mathbf {G}}_{gg}^{*}$ will be close to ${\mathbf {G}}_{gg}$. Regardless of the size of *s*, the resulting inverse is sparse because the sub-matrix corresponding to ${\mathbf {G}}_{nn}$ in the inverse has non-zero elements only on the diagonal. If the core group is chosen such that ${\mathbf {G}}_{cc}$ has rank less than *r* the rank of ${\mathbf {G}}_{gg}$, the matrix ${\mathbf {D}}$ will not be null, but as can be seen by examining Eq. (2) and demonstrated in the numerical example, it is not likely to be diagonal as assumed in the Apy algorithm. In this case the inverse computed by the Apy algorithm is the inverse of:

17 $$\begin{aligned} {\mathbf {G}}^{*}=\left[ \begin{array}{cc} {\mathbf {G}}_{cc}&\quad {\mathbf {G}}_{cc}{\mathbf {P}}^{\prime}\\ {{\mathbf {P}}}{{\mathbf {G}}}_{cc} & \quad {{\mathbf {P}}}{{\mathbf {G}}}_{cc}\text{ P }^{\prime}+\text {diag(}{\mathbf {D}}) \end{array}\right] , \end{aligned}$$

where $\text {diag}(\text{ D) }$ sets all the off-diagonal elements of ${\mathbf {D}}$ to zero, thus always leading to an approximation for that choice of core. Also, when ${\mathbf {G}}_{gg}$ is blended with ${\mathbf {A}}, {\mathbf {D}}$ will generally not be diagonal (see Additional file 3), and the inverse obtained in Apy is of ${\mathbf {G}}^{*}$ given by (17), where the off-diagonal elements of ${\mathbf {D}}$ have been set to zero.

### Numerical example

A small example with seven animals is used to illustrate the calculation of GBLUP and Apy-GBLUP. The pedigree for the seven animals is in Table 1. Genotype covariates coded as −1, 0, 1 at four loci are in Table 2. Julia scripts and results for GBLUP by strategies I to IV and for Apy-GBLUP are in Additional file 1. Only the calculations by strategy III and by Apy-GBLUP are described below.

**Table 1:** Pedigree for numerical example

Animal	Sire	Dam	PV	BV	EBV
1	0	0	99.25	−0.25	0.14
2	0	0	97.92	−0.94	−0.95
3	0	0	103.2	1.12	1.09
4	1	2	99.39	−1.01	−0.69
5	1	2	102.03	0.79	0.25
6	1	3	100.59	0.18	0.14
7	1	3	101.7	1.55	1.08

PV, BV and EBV are the phenotypic values, breeding values and the BLUPs of the BV

**Table 2:** Genotype covariates at four loci

Animal	Locus 1	Locus 2	Locus 3	Locus 4
1	0.0	0.0	−1.0	0.0
2	−1.0	1.0	0.0	0.0
3	1.0	0.0	−1.0	0.0
4	−1.0	0.0	0.0	1.0
5	0.0	1.0	0.0	1.0
6	0.0	1.0	−1.0	0.0
7	1.0	1.0	−1.0	0.0

#### Strategy III

The first step in this approach is to reorder the rows of ${\mathbf {M}}_{g}$ such that the first *r* rows are linearly independent, where *r* is the rank of ${\mathbf {M}}_{g}$. As described below, this can be done using Gaussian elimination with pivoting on ${\mathbf {M}}_{g}$ to transform it to row echelon form, where all elements below the diagonal are zero. Starting in row $i=1$, zeros are obtained below the diagonal by subtracting a multiple of row *i* from each subsequent row. Before doing these row operations to obtain zeros under the diagonal, the element with the largest absolute value is located in the sub-matrix comprising all rows below row $i-1$ and all columns to the right of column $i-1$. Then by swapping rows and columns, this element is moved to the $i^{\text {th}}$ diagonal. If the element with the largest absolute value is zero, Gaussian elimination is terminated. The rank of the matrix is the number of non-zero diagonals in the transformed matrix, and the rows used for Gaussian elimination provide a maximal set of linearly independent rows.

When Gaussian elimination was applied to genotype covariates in Table 2, the resulting matrix is in Table 3. All four diagonals of this matrix are non-zero, and so ${\mathbf {M}}_{g}$ has a rank equal to four. As a result of swapping rows, the rows were ordered as 2, 7, 1, 4, 5, 6, 3, where rows 2, 7, 1 and 4 were used for Gaussian elimination. Thus, these four rows are a linearly independent set, and they were taken to form ${\mathbf {M}}_{g_{i}}$ and rows 5, 6, and 3 were taken to form ${\mathbf {M}}_{g_{d}}.$ The genomic relationship matrix that was constructed using the reordered genotype covariates is in Table 4. The upper-left, $4\times 4$ sub matrix of this relationship matrix, denoted ${\mathbf {G}}_{g_{i}g_{i}}$, gives the relationships for individuals 2, 7, 1, and 4, and it is non-singular because the genotype covariates for these four individuals are linearly independent. Now, the matrix ${\mathbf {L}}^{\prime}$ can be calculated using (3), and is in Table 5.

**Table 3:** Genotype matrix transformed to row echelon form by Gaussian elimination with pivoting

−1.0	1.0	0.0	0.0
0.0	2.0	−1.0	0.0
0.0	0.0	−1.0	0.0
0.0	0.0	0.0	1.0
0.0	0.0	0.0	0.0
0.0	0.0	0.0	0.0
0.0	0.0	0.0	0.0

**Table 4:** Genomic relationship matrix

0.5	0.0	0.0	0.25	0.25	0.25	−0.25
0.0	0.75	0.25	−0.25	0.25	0.5	0.5
0.0	0.25	0.25	0.0	0.0	0.25	0.25
0.25	−0.25	0.0	0.5	0.25	0.0	−0.25
0.25	0.25	0.0	0.25	0.5	0.25	0.0
0.25	0.5	0.25	0.0	0.25	0.5	0.25
−0.25	0.5	0.25	−0.25	0.0	0.25	0.5

**Table 5:** The matrix ${\mathbf {L}}^{\prime}$ that relates ${\mathbf {M}}_{g_{i}}$ to ${\mathbf {M}}_{g_{d}}$ as ${\mathbf {M}}_{g_{d}}={\mathbf {L}}^{\prime}{\mathbf {M}}_{g_{i}}$

0.0	1.0	−1.0	1.0
0.5	0.5	0.5	0.0
−0.5	0.5	0.5	0.0

The last three rows of the matrix ${\mathbf {G}}_{gg}$ of genomic relationships in Table 4 can be written as a linear combination of the first four rows as shown in (2), by using the ${\mathbf {L}}^{\prime}$ matrix in Table 5. Thus, breeding values for individuals 5, 6, and 3 can be written as:

$$\begin{aligned} {\mathbf {u}}_{g_{d}}={\mathbf {L}}^{\prime}{\mathbf {u}}_{g_{i}}, \end{aligned}$$

where ${\mathbf {u}}_{g_{i}}$ is the vector of breeding values for individuals 2, 7, 1, and 4. Now, the phenotypes for these seven individuals are modeled in terms of ${\mathbf {u}}_{g_{i}}$, which has a non-singular covariance matrix proportional to ${\mathbf {G}}_{g_{i}g_{i}}.$ All seven individuals in this example have one phenotypic value, and so assuming that the vector $\varvec{\beta }$ of fixed effects contains a single element for the overall mean, the matrix ${\mathbf {X}}$ for this example is equal to a vector of seven 1s and ${\mathbf {Z}}$ is equal to an identity matrix of order seven. It follows that $${\mathbf {W}}=\begin{bmatrix}{\mathbf {I}}\\ {\mathbf {L}}^{\prime} \end{bmatrix}$$ for ${\mathbf {L}}^{\prime}$ in Table 5. The MME to fit the overall mean ($\mu )$ and the breeding values ${\mathbf {u}}_{g_{i}}$ are in Table 6, where a value of 1.0 was used for $\lambda =\frac{\sigma _{e}^{2}}{\sigma _{u}^{2}}.$ BLUP of ${\mathbf {u}}_{g}$ is obtained as $\hat{{\mathbf {u}}}_{g}={\mathbf {W}}\hat{{\mathbf {u}}}_{g_{i}}$. Results for strategies I through IV are in Additional file 1, and they are all identical, as expected. The condition numbers of the left-hand-side of the MME for strategies II through IV were 10.9, 11.3, and 6.8, demonstrating the improved condition of the MME obtained by fitting a RQ transformed vector of breeding values.

**Table 6:** Mixed model equations for $\mu$ and ${\mathbf {u}}_{g_{i}}$

	$\mu$	$u_{2}$	$u_{7}$	$u_{1}$	$u_{4}$
$\mu$	7.0	1.0	3.0	1.0	2.0
$u_{1}$	1.0	4.5	−1.0	1.0	−2.0
$u_{7}$	3.0	−1.0	5.5	−3.5	3.0
$u_{1}$	1.0	1.0	−3.5	9.5	−3.0
$u_{4}$	2.0	−2.0	3.0	−3.0	6.0
rhs	704.08	96.62	305.62	99.12	201.42
sol	100.43	−0.95	1.08	0.14	−0.69

The last two rows give the right-hand-side and the solutions of the equations

#### Apy-GBLUP

Here, we can see that if animals 2, 7, 1, and 4 are used as the core group, the Apy property is met because the last three rows of ${\mathbf {G}}_{gg}$, which correspond to the animals in the noncore group, can be written as a linear combination of the first four rows, which correspond to the animals in the core group, using the ${\mathbf {L}}^{\prime}$ matrix in Table 5 (see Additional file 1). Equation (2) shows that this property also holds for the columns of ${\mathbf {G}}_{gg}$, where the last three columns of ${\mathbf {G}}_{gg}$ can be written as a linear combination of the first four columns. In this case, the matrix ${\mathbf {D}}$, the inverse of which is needed in the Apy algorithm, is null (Additional file 1). In order to proceed with the Apy algorithm, we set ${\mathbf {D}}={\mathbf {I}}s$ for a small value of *s* such as 0.0001. The inverse that is obtained from equation (16) will now be sparse because the sub-matrix corresponding to ${\mathbf {G}}_{nn}$ in the inverse is diagonal. Inverting a modified ${\mathbf {G}}_{gg}$ matrix, ${\mathbf {G}}_{gg}^{*}$, by adding *s* to the diagonals of ${\mathbf {G}}_{gg}$ corresponding to the animals in the noncore group gives the same result (Additional file 1). Setting up and solving the MME for $\mu$ and ${\mathbf {u}}_{g}$ assuming $\text {Var}({\mathbf {u}}_{g})={\mathbf {G}}_{gg}^{*}\sigma _{u}^{2}$ give results that are approximate but very close in this instance to the exact BLUP results obtained by strategies I through IV (Additional file 1), but the condition number of these MME was 56,548, which indicates that they are ill-conditioned relative to those for strategies II through IV. However, if individuals 2, 7, and 1 are chosen as the core animals, the Apy property does not hold. In that case, the last four rows of ${\mathbf {G}}_{gg}$ cannot be written as a linear combination of the first three rows (Additional file 2). Furthermore, the matrix ${\mathbf {D}}$ computed by using equation (15) is not diagonal (Additional file 2). Now, the matrix ${\mathbf {G}}_{gg}^{*}$ that is inverted in the Apy algorithm deviates substantially from ${\mathbf {G}}_{gg}$, and as a result, solving the MME for $\mu$ and ${\mathbf {u}}_{g}$ assuming $\text {Var}({\mathbf {u}}_{g})={\mathbf {G}}_{gg}^{*}\sigma _{u}^{2}$ gives results that are substantially different from the exact BLUP (Additional file 2).

Recent publications [12, 13, 18] in which the Apy algorithm was applied to obtain a matrix portrayed as the inverse of the genomic relationship matrix use $0.95{\mathbf {G}}_{gg}+0.05{\mathbf {A}}$ rather than the singular ${\mathbf {G}}_{gg}$. This approach applied to the example gives a solution that is neither the same as the exact solution obtained using any of the strategies I to IV (Additional file 1), nor the exact solution to the MME constructed with the blended genomic relationship matrix. However, the condition number of these equations was 62.1, which is much better than that obtained without blending but poorer than with strategies II through IV.

### Exact single-step GBLUP when G_gg_ is singular

Single-step GBLUP (SS-GBLUP) was proposed [23, 24] to obtain genomic evaluations when genotypes are not available on all animals.

#### Strategy III

Let ${\mathbf {u}}_{g}$ denote the breeding values of animals with genotypes and ${\mathbf {u}}_{m}$ denote the breeding values of those without genotypes. Now, the mixed linear model for SSGBLUP can be written as:

$$\begin{aligned} {\mathbf {y}}={\mathbf {X}}\varvec{\beta }+{\mathbf {Z}}\left[ \begin{array}{c} {\mathbf {u}}_{m}\\ {\mathbf {u}}_{g} \end{array}\right] +{\mathbf {e}}. \end{aligned}$$

It is convenient to similarly partition the vector of phenotypic values as $${\mathbf {y}}=\left[ \begin{array}{c} {\mathbf {y}}_{m} \\ {\mathbf {y}}_{g} \end{array}\right]$$, where ${\mathbf {y}}_{m}$ are phenotypic values from animals that were not genotyped and ${\mathbf {y}}_{g}$ are from animals that were genotyped. However, because ${\mathbf {G}}_{gg}$ is singular, ${\mathbf {u}}_{g}$ is written as in Eq. (11) in terms of ${\mathbf {u}}_{g_{i}}$, and then the model becomes:

18 $$\begin{aligned} \left[ \begin{array}{c} {\mathbf {y}}_{m}\\ {\mathbf {y}}_{g} \end{array}\right] &= {\mathbf {X}}\varvec{\beta }+{\mathbf {Z}}\left[ \begin{array}{cc} {\mathbf {I}} &{} {\mathbf {0}}\\ {\mathbf {0}} &{} {\mathbf {S}} \end{array}\right] \left[ \begin{array}{c} {\mathbf {u}}_{m}\\ {\mathbf {u}}_{g_{i}} \end{array}\right] +{\mathbf {e}}\nonumber \\ & = {\mathbf {X}}\varvec{\beta }+{\mathbf {W}}_{r}\mathbf {u_{r}}+{\mathbf {e}}, \end{aligned}$$

where $${\mathbf {S}}=\left[ \begin{array}{c} {\mathbf {I}}\\ {\mathbf {L}}^{\prime} \end{array}\right] , {\mathbf {W}}_{r}={\mathbf {Z}}\left[ \begin{array}{cc} {\mathbf {I}} &{} {\mathbf {0}}\\ {\mathbf {0}} &{} {\mathbf {S}} \end{array}\right]$$, and $${\mathbf {u}}_{r}=\left[ \begin{array}{c} {\mathbf {u}}_{m}\\ {\mathbf {u}}_{g_{i}} \end{array}\right]$$. The MME that correspond to (18) are:

19 $$\begin{aligned} \left[ \begin{array}{cc} {\mathbf {X}}^{\prime}{\mathbf {X}} &{} {\mathbf {X}}^{\prime}{\mathbf {W}}_{r}\\ {\mathbf {W}}_{r}^{\prime}{\mathbf {X}} &{} {\mathbf {W}}_{r}^{\prime}{\mathbf {W}}_{r}+{\mathbf {H}}_{r}^{-1} \end{array}\right] \left[ \begin{array}{c} \hat{\varvec{\beta }}\\ \hat{{\mathbf {u}}}_{r} \end{array}\right] =\left[ \begin{array}{c} {\mathbf {X}}^{\prime}{\mathbf {y}}\\ {\mathbf {W}}_{r}^{\prime}{\mathbf {y}} \end{array}\right] , \end{aligned}$$

where

$$\begin{aligned} {\mathbf {H}}_{r}=\text {Var}({\mathbf {u}}_{r}). \end{aligned}$$

To obtain ${\mathbf {H}}_{r}^{-1}$, as in [23], ${\mathbf {u}}_{m}$ is written as:

$$\begin{aligned} {\mathbf {u}}_{m} &= {\mathbf {A}}_{mg}{\mathbf {A}}_{gg}^{-1}{\mathbf {u}}_{g}+{\mathbf {u}}_{m}-{\mathbf {A}}_{mg}{\mathbf {A}}_{gg}^{-1}{\mathbf {u}}_{g}\\ &= {\mathbf {A}}_{mg}{\mathbf {A}}_{gg}^{-1}{\mathbf {u}}_{g}+\mathbf {\varvec{\epsilon }}\\ &= -({\mathbf {A}}^{mm})^{-1}{\mathbf {A}}^{mg}{\mathbf {u}}_{g}+\mathbf {\varvec{\epsilon }}, \end{aligned}$$

where, in the last line, we have used the identity: ${\mathbf {A}}_{mg}{\mathbf {A}}_{gg}^{-1}=-({\mathbf {A}}^{mm})^{-1}{\mathbf {A}}^{mg}$ . Now, ${\mathbf {u}}_{r}$ is written in terms of ${\mathbf {u}}_{g_{i}}$ and $\varvec{\epsilon }$ as:

$$\begin{aligned} {\mathbf {u}}_{r} &= \left[ \begin{array}{cc} {\mathbf {I}} &{} -({\mathbf {A}}^{mm})^{-1}{\mathbf {A}}^{mg}{\mathbf {S}}\\ {\mathbf {0}} &{} {\mathbf {I}} \end{array}\right] \left[ \begin{array}{l} \varvec{\epsilon }\\ {\mathbf {u}}_{g_{i}} \end{array}\right] \\ &= {\mathbf {T}}\left[ \begin{array}{l} \varvec{\epsilon }\\ {\mathbf {u}}_{g_{i}} \end{array}\right] , \end{aligned}$$

where ${\mathbf {u}}_{g}={{\mathbf {S}}}{{\mathbf {u}}}_{g_{i}}$ and $${\mathbf {T}}=\left[ \begin{array}{cc} {\mathbf {I}} &{} -({\mathbf {A}}^{mm})^{-1}{\mathbf {A}}^{mg}{\mathbf {S}}\\ {\mathbf {0}} &{} {\mathbf {I}} \end{array}\right]$$, and ${\mathbf {H}}_{r}$ is written as

$$\begin{aligned} {\mathbf {H}}_{r}={\mathbf {T}}\text {Var}\left( \left[ \begin{array}{l} \varvec{\epsilon }\\ {\mathbf {u}}_{g_{i}} \end{array}\right] \right) \mathbf T ^{\prime}. \end{aligned}$$

Following [23], $\text {Var}(\varvec{\epsilon })=({\mathbf {A}}^{mm})^{-1}\sigma _{a}^{2}, \text {Var}({\mathbf {u}}_{g_{i}})={\mathbf {G}}_{g_{i}g_{i}}\sigma _{u}^{2}$, and $\text {Cov}(\varvec{\epsilon },{\mathbf {u}}_{g_{i}})={\mathbf {0}}$. Unlike in [23], two variance components $\sigma _{a}^{2}$ and $\sigma _{u}^{2}$ are used here, where $\sigma _{a}^{2}$ is the additive genetic variance and $\sigma _{u}^{2}=k\sigma _{\alpha }^{2}$ stems from the prior used for the marker effects ($\varvec{\alpha })$, and its relationship to the genetic variance may not be straightforward [25]. Finally, ${\mathbf {H}}_{r}^{-1}$ is as follows:

$$\begin{aligned} {\mathbf {H}}_{r}^{-1} &= ({\mathbf {T}}^{\prime})^{-1}\left[ \begin{array}{cc} {\mathbf {A}}^{mm}\frac{1}{\sigma _{a}^{2}} &\quad {\mathbf {0}} \\ {\mathbf {0}} & \quad {\mathbf {G}}_{g_{i}g_{i}}^{-1}\frac{1}{\sigma _{u}^{2}} \end{array}\right] {\mathbf {T}}^{-1} \\ &= \left[ \begin{array}{cc} {\mathbf {I}} & \quad {\mathbf {0}}\\ {\mathbf {S}}^{\prime}{\mathbf {A}}^{gm}({\mathbf {A}}^{mm})^{-1} & \quad {\mathbf {I}} \end{array}\right] \left[ \begin{array}{cc} {\mathbf {A}}^{mm}\frac{1}{\sigma _{a}^{2}} &\quad {\mathbf {0}}\\ {\mathbf {0}} & \quad {\mathbf {G}}_{g_{i}g_{i}}^{-1}\frac{1}{\sigma _{u}^{2}} \end{array}\right] \left[ \begin{array}{cc} {\mathbf {I}} & \quad ({\mathbf {A}}^{mm})^{-1}{\mathbf {A}}^{mg}{\mathbf {S}}\\ {\mathbf {0}} & \quad {\mathbf {I}} \end{array}\right] \\ &= \left[ \begin{array}{cc} {\mathbf {A}}^{mm}\frac{1}{\sigma _{a}^{2}} & \quad {\mathbf {A}}^{mg}{\mathbf {S}}\frac{1}{\sigma _{a}^{2}}\\ {\mathbf {S}}^{\prime}{\mathbf {A}}^{gm}\frac{1}{\sigma _{a}^{2}} &\quad {\mathbf {Q}}\frac{1}{\sigma _{a}^{2}}+{\mathbf {G}}_{g_{i}g_{i}}^{-1}\frac{1}{\sigma _{u}^{2}} \end{array}\right] , \end{aligned}$$

where

$$\begin{aligned} {\mathbf {Q}}={\mathbf {S}}^{\prime}{\mathbf {A}}^{gm}({\mathbf {A}}^{mm})^{-1}{\mathbf {A}}^{mg}{\mathbf {S}}. \end{aligned}$$

Following [26], the MME given in equation (19) can be augmented to avoid the expression involving $({\mathbf {A}}^{mm})^{-1}$ in ${\mathbf {Q}}$. To do so, equation (19) is first rewritten to show the partitions for ${\mathbf {u}}_{m}$ and ${\mathbf {u}}_{g_{i}}$. Note that the matrix ${\mathbf {Z}}$ has the following form:

$$\begin{aligned} {\mathbf {Z}}=\left[ \begin{array}{cc} {\mathbf {Z}}_{m} &{} {\mathbf {0}}\\ {\mathbf {0}} &{} {\mathbf {Z}}_{g} \end{array}\right] , \end{aligned}$$

where ${\mathbf {Z}}_{m}$ relates ${\mathbf {y}}_{m}$ to ${\mathbf {u}}_{m}$ and ${\mathbf {Z}}_{g}$ relates ${\mathbf {y}}_{g}$ to ${\mathbf {u}}_{g}$. Then, ${\mathbf {W}}_{r}$ can be partitioned as:

$$\begin{aligned} {\mathbf {W}}_{r}&=\left[ \begin{array}{cc} {\mathbf {Z}}_{m} & {\mathbf {0}}\\ {\mathbf {0}} &{} {\mathbf {Z}}_{g} \end{array}\right] \left[ \begin{array}{cc} {\mathbf {I}} &{} {\mathbf {0}}\\ {\mathbf {0}} &{} {\mathbf {S}} \end{array}\right] \\&=\left[ \begin{array}{cc} {\mathbf {Z}}_{m} &{} {\mathbf {0}}\\ {\mathbf {0}} &{} {\mathbf {W}}_{g_{i}} \end{array}\right] , \end{aligned}$$

where ${\mathbf {W}}_{g_{i}}={\mathbf {Z}}_{g}{\mathbf {S}}$. Now the MME that show the partitions for ${\mathbf {u}}_{m}$ and ${\mathbf {u}}_{g_{i}}$ are:

20 $$\begin{aligned} \left[ \begin{array}{ccc} {\mathbf {X}}^{\prime}{\mathbf {X}} & \quad {\mathbf {X}}_{m}^{\prime}{\mathbf {Z}}_{m} & \quad {\mathbf {X}}_{g}^{\prime}{\mathbf {W}}_{g_{i}}\\ {\mathbf {Z}}^{\prime}_{m}{\mathbf {X}}_{m} &\quad {\mathbf {Z}}^{\prime}_{m}{\mathbf {Z}}_{m}+{\mathbf {A}}^{mm}\frac{\sigma _{e}^{2}}{\sigma _{a}^{2}} & \quad {\mathbf {A}}^{mg}{\mathbf {S}}\frac{\sigma _{e}^{2}}{\sigma _{a}^{2}}\\ {\mathbf {W}}^{\prime}_{g_{i}}{\mathbf {X}}_{g} &\quad {\mathbf {S}}^{\prime}{\mathbf {A}}^{gm}\frac{\sigma _{e}^{2}}{\sigma _{a}^{2}} & \quad{\mathbf {W}}^{\prime}_{g_{i}}{\mathbf {W}}_{g_{i}}+{\mathbf {G}}_{g_{i}g_{i}}^{-1}\frac{\sigma _{e}^{2}}{\sigma _{u}^{2}}+{\mathbf {Q}}\frac{\sigma _{e}^{2}}{\sigma _{a}^{2}} \end{array}\right] \left[ \begin{array}{c} \hat{\varvec{\beta }}\\ {\hat{\mathbf{u}}}_{m}\\ \hat{{\mathbf {u}}}_{g_{i}} \end{array}\right] =\left[ \begin{array}{c} {\mathbf {X}}^{\prime}{\mathbf {y}}\\ {\mathbf {Z}}^{\prime}_{m}{\mathbf {y}}_{m}\\ {\mathbf {W}}^{\prime}_{g_{i}}{\mathbf {y}}_{g} \end{array}\right] , \end{aligned}$$

where ${\mathbf {X}}_{m}$ and ${\mathbf {X}}_{g}$ are partitions of ${\mathbf {X}}$ corresponding to ${\mathbf {y}}_{m}$ and ${\mathbf {y}}_{g}$. Consider now the following augmented MME:

21 $$\begin{aligned} \left[ \begin{array}{cccc} {\mathbf {X}}^{\prime}{\mathbf {X}} &\quad {\mathbf {X}}_{m}^{\prime}{\mathbf {Z}}_{m} & \quad {\mathbf {X}}_{g}^{\prime}{\mathbf {W}}_{g_{i}} & \quad {\mathbf {0}}\\ {\mathbf {Z}}^{\prime}_{m}{\mathbf {X}}_{m} & \quad {\mathbf {Z}}^{\prime}_{m}{\mathbf {Z}}_{m}+{\mathbf {A}}^{mm}\frac{\sigma _{e}^{2}}{\sigma _{a}^{2}} &\quad {\mathbf {A}}^{mg}{\mathbf {S}}\frac{\sigma _{e}^{2}}{\sigma _{a}^{2}} & \quad {\mathbf {0}}\\ {\mathbf {W}}^{\prime}_{g_{i}}{\mathbf {X}}_{g} & \quad {\mathbf {S}}^{\prime}{\mathbf {A}}^{gm}\frac{\sigma _{e}^{2}}{\sigma _{a}^{2}} & \quad {\mathbf {W}}^{\prime}_{g_{i}}{\mathbf {W}}_{g_{i}}+{\mathbf {G}}_{g_{i}g_{i}}^{-1}\frac{\sigma _{e}^{2}}{\sigma _{u}^{2}} & \quad -{\mathbf {S}}^{\prime}{\mathbf {A}}^{gm}\frac{\sigma _{e}^{2}}{\sigma _{a}^{2}}\\ {\mathbf {0}} & \quad {\mathbf {0}} & \quad -{\mathbf {A}}^{mg}{\mathbf {S}}\frac{\sigma _{e}^{2}}{\sigma _{a}^{2}} & \quad -{\mathbf {A}}^{mm}\frac{\sigma _{e}^{2}}{\sigma _{a}^{2}} \end{array}\right] \left[ \begin{array}{c} \hat{\varvec{\beta }}\\ {\hat{\mathbf{u}}}_{m}\\ \hat{{\mathbf {u}}}_{g_{i}}\\ {\mathbf {c}} \end{array}\right] =\left[ \begin{array}{c} {\mathbf {X}}^{\prime}{\mathbf {y}}\\ {\mathbf {Z}}^{\prime}_{m}{\mathbf {y}}_{m}\\ {\mathbf {W}}^{\prime}_{g_{i}}{\mathbf {y}}_{g}\\ {\mathbf {0}} \end{array}\right] . \end{aligned}$$

These equations do not have ${\mathbf {Q}}$ in them, and so they may be easier to construct. However, the left-hand-side is not positive definite and it has been reported that these equations are poorly conditioned [26]. Elimination of ${\mathbf {c}}$ from Eq. (21) results in equation (20), and thus, solutions for $\hat{\varvec{\beta }}, {\hat{\mathbf{u}}}_{m}$ and for $\hat{{\mathbf {u}}}_{g_{i}}$ from Eq. (21) are identical to those from Eq. (20).

#### Strategy IV

The model for SSGBLUP can also be formulated in terms of ${\mathbf {v}}$ as:

22 $$\begin{aligned} \left[ \begin{array}{c} {\mathbf {y}}_{m}\\ {\mathbf {y}}_{g} \end{array}\right] ={\mathbf {X}}\varvec{\beta }+{\mathbf {Z}}\left[ \begin{array}{cc} {\mathbf {I}} &{} {\mathbf {0}}\\ {\mathbf {0}} &{} {\mathbf {R}} \end{array}\right] \left[ \begin{array}{c} {\mathbf {u}}_{m}\\ {\mathbf {v}} \end{array}\right] +{\mathbf {e}}, \end{aligned}$$

and the MME corresponding to model (22) are:

23 $$\begin{aligned} \left[ \begin{array}{ccc} {\mathbf {X}}^{\prime}{\mathbf {X}} &\quad {\mathbf {X}}_{m}^{\prime}{\mathbf {Z}}_{m} & \quad {\mathbf {X}}_{g}^{\prime}{\mathbf {W}}_{v}\\ {\mathbf {Z}}^{\prime}_{m}{\mathbf {X}}_{m} &\quad {\mathbf {Z}}^{\prime}_{m}{\mathbf {Z}}_{m}+{\mathbf {A}}^{mm}\frac{\sigma _{e}^{2}}{\sigma _{a}^{2}} & \quad {\mathbf {A}}^{mg}{\mathbf {R}}\frac{\sigma _{e}^{2}}{\sigma _{a}^{2}}\\ {\mathbf {W}}^{\prime}_{v}{\mathbf {X}}_{g} & \quad {\mathbf {R}}^{\prime}{\mathbf {A}}^{gm}\frac{\sigma _{e}^{2}}{\sigma _{a}^{2}} &\quad {\mathbf {W}}^{\prime}_{v}{\mathbf {W}}_{v}+{\mathbf {I}}\frac{\sigma _{e}^{2}}{\sigma _{\alpha }^{2}}+{\mathbf {Q}}_{v}\frac{\sigma _{e}^{2}}{\sigma _{a}^{2}} \end{array}\right] \left[ \begin{array}{c} \hat{\varvec{\beta}} \\ {\hat{\mathbf{u}}}_{m}\\ \hat{{\mathbf {v}}} \end{array}\right] =\left[ \begin{array}{c} {\mathbf {X}}^{\prime}{\mathbf {y}}\\ {\mathbf {Z}}^{\prime}_{m}{\mathbf {y}}_{m}\\ {\mathbf {W}}^{\prime}_{v}{\mathbf {y}}_{g} \end{array}\right] , \end{aligned}$$

where $${\mathbf {W}}_{v}={\mathbf {Z}}_{g}{\mathbf {R}}$$ and $${\mathbf {Q}}_{v}={\mathbf {R}}^{\prime}{\mathbf {A}}^{gm}({\mathbf {A}}^{mm})^{-1}{\mathbf {A}}^{mg}{\mathbf {R}}$$.

#### Comparison to Apy-SSGBLUP

The SSGBLUP method given in [23] requires computing the inverse of the matrix ${\mathbf {G}}_{gg}$ of genomic relationships and of the matrix ${\mathbf {A}}_{gg}$ of additive relationships for the genotyped animals. At the time those papers were published, $N_{g}$ was typically smaller than the number of markers so that ${\mathbf {G}}_{gg}$ was relatively small and of full rank. Since then $N_{g}$ has greatly increased in most livestock applications. Computational effort in matrix manipulation is determined by the number of non-zero coefficients and these increase as $N_{g}$ increases. To fully store a dense matrix of order one million in single precision requires about 4 TB. Therefore, it would be advantageous to have a sparse representation of all the large matrices involved in the MME.

Furthermore, the matrix ${\mathbf {G}}_{gg}$ is singular when $N_{g}>k$ and thus cannot be inverted when more animals than the number of SNPs have been genotyped. This suggests that there should be a sparse representation of ${\mathbf {G}}_{gg}$. Suppose ${\mathbf {G}}_{gg}$ has rank *r* and it is ordered such that the first *r* rows are linearly independent. Then, the sub-matrix of the first *r* rows and columns of ${\mathbf {G}}_{gg}$ denoted ${\mathbf {G}}_{cc}$ gives the genomic relationships among the *r* core animals of the Apy algorithm, and that sub-matrix is nonsingular. The remaining $n-r$ animals are referred to as noncore and their genomic relationship matrix is denoted ${\mathbf {G}}_{nn}$. When the genomic relationship matrix has not been blended, a nonsingular matrix ${\mathbf {G}}^{*}$ can be obtained by adding a small value to the diagonals of ${\mathbf {G}}_{gg}$ for the animals in the noncore group, and in the inverse of ${\mathbf {G}}^{*}$, the sub-matrix corresponding to ${\mathbf {G}}_{nn}$ will be diagonal. This exact inverse of that particular ${\mathbf {G}}^{*}$ can be obtained efficiently using the Apy algorithm given in [9]. When the genomic relationship matrix is blended with ${\mathbf {A}},$ the matrix resulting from the Apy algorithm is the exact inverse of the ${\mathbf {G}}^{*}$ where all the off-diagonals of ${\mathbf {D}}$ have been ignored. That matrix may or may not be a close approximation of the blended genomic relationship matrix depending on the size of the core, the particular animals chosen for the core, the relationship among noncore animals and the relationship between core and noncore animals. Regardless of the form of the matrix the Apy algorithm is applied to, the resultant inverse is sparse.

The MME for Apy-SSGBLUP, which includes the inverse of ${\mathbf {G}}^{*}$, are:

24 $$\begin{aligned} \left[ \begin{array}{ccc} {\mathbf {X}}^{\prime}{\mathbf {X}} & \quad {\mathbf {X}}_{m}^{\prime}{\mathbf {Z}}_{m} &\quad {\mathbf {X}}_{g}^{\prime}{\mathbf {Z}}_{g}\\ {\mathbf {Z}}^{\prime}_{m}{\mathbf {X}}_{m} &\quad {\mathbf {Z}}^{\prime}_{m}{\mathbf {Z}}_{m}+{\mathbf {A}}^{mm}\frac{\sigma _{e}^{2}}{\sigma _{a}^{2}} & \quad {\mathbf {A}}^{mg}\frac{\sigma _{e}^{2}}{\sigma _{a}^{2}}\\ {\mathbf {Z}}^{\prime}_{g}{\mathbf {X}}_{g} & \quad {\mathbf {A}}^{gm}\frac{\sigma _{e}^{2}}{\sigma _{a}^{2}} & \quad {\mathbf {Z}}^{\prime}_{g}{\mathbf {Z}}_{g}+[({\mathbf {G}}^{*})^{-1}-{\mathbf {A}}_{gg}^{-1}]\frac{\sigma _{e}^{2}}{\sigma _{u}^{2}} \end{array}\right] \left[ \begin{array}{c} \hat{\varvec{\beta }}\\ {\hat{\mathbf{u}}}_{m}\\ \hat{{\mathbf {u}}}_{g} \end{array}\right] =\left[ \begin{array}{c} {\mathbf {X}}^{\prime}{\mathbf {y}}\\ {\mathbf {Z}}^{\prime}_{m}{\mathbf {y}}_{m}\\ {\mathbf {Z}}^{\prime}_{g}{\mathbf {y}}_{g} \end{array}\right] . \end{aligned}$$

In addition to the inverse ${\mathbf {G}}^{*}$, SSGBLUP requires the inverse of ${\mathbf {A}}_{gg}$. However, ${\mathbf {A}}_{gg}^{-1}$ is a dense matrix, and so subtracting it from the inverse of ${\mathbf {G}}^{*}$ will make the resultant matrix dense.

Part of the appeal of the Apy algorithm was to obtain a sparse representation of the MME for SSGBLUP. Accordingly, Misztal et al. [9] proposed that Apy could also be used to approximate the inverse of the nonsingular ${\mathbf {A}}_{gg}$. However, the nature of ${\mathbf {A}}_{gg}$ depends on the genotyping strategy such that genotyping unrelated individuals results in a diagonal ${\mathbf {A}}_{gg}$ whereas genotyping relatives results in non-zero off-diagonals between each related pair. If off-diagonal elements in the noncore sub matrix of ${\mathbf {A}}_{gg}$ are not well predicted by ${{\mathbf {P}}}{{\mathbf {G}}}_{cc}{\mathbf {P}}^{\prime}$, the Apy inverse can significantly depart from its true inverse as easily demonstrated by using an example (see Additional file 1). This means the adequacy of Apy applied to ${\mathbf {A}}_{gg}$ will depend on the pedigree structure, the nature of the genotyping strategy, and the choice of core group. Presumably, this inadequacy of Apy for inverting ${\mathbf {A}}_{gg}$ has been recognized because recent implementations [13] have adopted an alternative approach that is computationally more demanding than applying Apy to approximate the inverse of ${\mathbf {A}}_{gg}$. Rather than forming ${\mathbf {A}}_{gg}^{-1}$ prior to solving the MME, a partitioned matrix inverse result is used to calculate products such as ${\mathbf {A}}_{gg}^{-1}{\mathbf {x}}$ as ${\mathbf {A}}^{gg}{\mathbf {x}}-{\mathbf {A}}^{gm}{\mathbf {q}}$, where ${\mathbf {q}}$ is the solution to ${\mathbf {A}}^{mm}{\mathbf {q}}={\mathbf {A}}^{mg}{\mathbf {x}}$. This requires storing the sparse matrices ${\mathbf {A}}^{gg},{\mathbf {A}}^{mg}$ and the sparse Cholesky factors of ${\mathbf {A}}^{mm}$. Each PCG iteration involves a matrix product ${\mathbf {A}}_{gg}^{-1}{\mathbf {x}}$ for a different vector ${\mathbf {x}}$, which requires one forward and one backward triangular solve to obtain ${\mathbf {q}}$, two sparse matrix vector multiplications, and one vector subtraction.

Both the MME for Apy-SSGBLUP and that for SSGBLUP using strategy IV (SIV-SSGBLUP), include equations for the same fixed effects and the random effects corresponding to the breeding values of animals that were not genotyped. In the MME for Apy-SSGBLUP, there is an additional vector of random effects corresponding to the breeding values for animals that were genotyped, which comprises sub-vectors representing core and noncore animals. In contrast, the MME for SSGBLUP using strategy IV contains a vector of random effects that is not larger than *k* regardless of the number of animals genotyped. If the core size in Apy-SSGBLUP was chosen to be *k*, Eq. (24) would contain an additional random effect of order equal to the number of noncore animals compared to Eq. (23), and this number increases with the number of animals genotyped.

Given a core of *k* animals, both MME contain a dense $k\times k$ matrix on the diagonal. Both MME contain the same sparse block on the diagonal for non-genotyped animals. Comparing the upper off-diagonals of the two sets of symmetric MME, that for Apy-SSGBLUP has the sparse ${\mathbf {A}}^{mg}$ matrix whereas SIV-SSGBLUP has the product of that $N_{m}\times N_{g}$ matrix with the mostly dense $N_{g}\times k$ matrix ${\mathbf {R}}$. Rather than forming the dense $N_{m}\times k$ product, matrix computations involving that matrix can be done more efficiently when $N_{g}<N_{m}$ in parts (e.g. ${\mathbf {A}}^{mg}{{\mathbf {R}}}{{\mathbf {x}}}={\mathbf {A}}^{mg}({{\mathbf {R}}}{{\mathbf {x}}})$) storing only ${\mathbf {A}}^{mg}$ and ${\mathbf {R}}$ in memory. The Apy-SSGBLUP MME contain on the upper diagonal a dense $N_{c}\times N_{n}$ block that does not appear in SIV-SSGBLUP and which increases in size as more animals are genotyped. The computation required to form the diagonal block of SIV-SSGBLUP involves computing $({\mathbf {A}}^{mm})^{-1}{\mathbf {A}}^{mg}\mathbf {r_{i}}$, where ${\mathbf {r}}_{i}$ is column *i* of ${\mathbf {R}}$. This calculation is virtually identical to the computation of ${\mathbf {A}}_{gg}^{-1}{\mathbf {x}}$ in Apy-SSGBLUP, but the former needs to be done for each genotyped animal once whereas the latter needs to be done for each PCG iteration.

## Discussion

When the number $N_{g}$ of genotyped animals is larger than the number *k* of marker covariates, the matrix ${\mathbf {G}}_{gg}$ of genomic relationships becomes singular. In this situation, we have shown here how to obtain exact GBLUP without any approximation from either Eqs. (13) or (14) of order $p+r$ or $p+k$, where $r\le k$ is the rank of ${\mathbf {G}}_{cc}$. The MME given by Eq. (9) can also be used to obtain GBLUP without approximation, but these asymmetric MME are of order $p+N_{g}$. When more individuals are genotyped and $N_{g}$ grows, the order of those MME (9) also grows. In contrast, the order of the MME presented here (Eqs. 13 and 14) will remain constant even as $N_{g}$ grows.

An alternative to these exact GBLUP calculations is used in Apy-GBLUP. Here, the pedigree is divided into two groups of animals: the core group and the noncore group. We have shown here that the inverse computed in the Apy algorithm is for a modified genomic relationship matrix, where the sub-matrix ${\mathbf {G}}_{nn}$ of genomic relationships among the noncore group of animals is replaced by ${{\mathbf {P}}}{{\mathbf {G}}}_{cc}{\text{P}}^{\prime}+\text{diag}({\mathbf {D}})$. If the core group is chosen such that the rank of ${\mathbf {G}}_{cc}$ is equal to the rank of ${\mathbf {G}}_{gg}, {\mathbf {D}}$ will be null and the Apy algorithm will fail. In that case, the diagonals of ${\mathbf {D}}$ can be set to some small value, but this can result in ill-conditioned MME as shown by the example in Additional file 1. The MME can be ill-conditioned even when ${\mathbf {D}}$ is not null but contains very small values on the diagonal. Although the MME for Apy-GBLUP will also grow with $N_{g}$, it contains a $N_{g_{d}}\times N_{g_{d}}$ block that is diagonal, and thus is very sparse.

The approach presented here can also be used to obtain exact SSGBLUP when some animals are not genotyped. In contrast to the Apy algorithm, the method presented here is never an approximation. In agreement with [10], “BVs of core individuals can all be written as linear combinations of effective SNP effects” when SNP effects fully explain the BV. In contrast to the claim in [10] that “BVs of noncore individuals depend approximately only on the BVs of the core individuals” we have shown that the BVs of noncore individuals are an exact linear function of the BVs of the core individuals when the rank of ${\mathbf {G}}_{cc}$ is equal to the rank of ${\mathbf {G}}_{gg}.$ This requires the core group to contain at least as many animals as the rank of ${\mathbf {G}}_{gg}.$ When the number of genotyped animals exceeds the number of markers, ${\mathbf {G}}_{gg}$ will be singular and its rank cannot be greater than the number of markers. Only when the rank of ${\mathbf {G}}_{cc}$ is less than the rank of ${\mathbf {G}}_{gg}$, will the “BVs of noncore individuals depend approximately only on the BVs of the core individuals”.

The Apy algorithm when applied to ${\mathbf {A}}_{gg}$ may or may not be a good approximation depending on the particular ${\mathbf {A}}_{gg}$. It will be exact for any core if genotyped animals are all unrelated as the matrix ${\mathbf {D}}$ is strictly diagonal.The quality of the approximation will erode with increases in the number of large-magnitude off-diagonal elements in ${\mathbf {D}}$. Demonstrating with real data that the Apy gives good results in one or more field data sets is no guarantee that it will perform well for all applications. This raises concerns that the same could be true for the application of Apy to the genomic relationship matrix. When the number of genotyped individuals increases and the number of core animals remains constant, there may be a large increase in the number of off-diagonal coefficients in ${\mathbf {D}}$. Those coefficients are ignored in the Apy algorithm, and the predictions approximated by Apy are expected to deviate further from the exact predictions as more coefficients are ignored. Thus, inference that the Apy algorithm based on 100,000 or 500,000 genotyped animals is appropriate cannot be extrapolated to similar data structures with a million or more animals genotyped.

If SNP effects do not fully explain the BV, an additional polygenic effect for all animals can be readily fitted in addition to ${\mathbf {u}}_{g_{i}}$, the breeding values explained by the markers for a subset of genotyped animals. Lourenco et al. [12] used default options of BLUP90IOD2, which means they blended ${\mathbf {G}}_{gg}$ with ${\mathbf {A}}_{22}$, and included competitive results from Apy compared to exact predictions obtained by direct inversion for various analyses with $N_{g}$ that were smaller than 52,000. Fragomeni et al. [11] limited their analyses to $N_{g}=100,000$ in order to allow direct inversion of a ${\mathbf {G}}_{gg}$ matrix based on 42,503 SNPs but do not mention whether blending was used. In the absence of blending, the rank of ${\mathbf {G}}_{gg}$ matrix could not exceed 42,503 and a direct inverse of ${\mathbf {G}}_{gg}$ does not exist. Pocrnic et al. [27] simulated genotypes on 75,000 individuals and blended ${\mathbf {G}}_{gg}$ with ${\mathbf {A}}_{gg}$. They showed that Apy exceeded the accuracy of exact ssGBLUP by direct inversion. Their QTL effects were simulated from a Gamma distribution, which creates a few loci with large effects. In those circumstances, methods such as BayesB typically outperform GBLUP [3], and Apy with a small set of core individuals may similarly benefit from reducing the dimension of the model. Masuda et al. [13] blended ${\mathbf {G}}_{gg}$ with ${\mathbf {A}}_{gg}$ to guarantee nonsingularity of the blended matrix with $N_{g}$ greater than 500,000. The exact inverse of that blended matrix will be a dense matrix of order $N_{g}$, which will make exact calculations computationally infeasible when $N_{g}$ exceeds about 150,000. This makes it impossible to compare the accuracy of Apy approximations to exact predictions using that approach. They show high correlations between approximations for different core definitions but the correlations between their approximations and the exact predictions are not known.

There are no published results demonstrating the comparative accuracy of Apy and the exact approach when $N_{G}$ is too large for direct inversion of ${\mathbf {G}}_{gg}.$ However, using the exact SSGBLUP calculations presented in this paper such a comparison is feasible, requiring only special computation for ${\mathbf {R}}$ and ${\mathbf {Q}}$ in the MME (23). Computation of the matrix ${\mathbf {Q}}$ involves the same calculations as required to impute genotypes for non-genotyped animals as presented in Fernando et al. [28]. The computation of **R** is straightforward and analogous to matrix ${\mathbf {P}}$ that is fundamental to computations in the Apy algorithm. Accordingly, we do not consider that the practical application of exact SSGBLUP will be any more difficult than implementation of Apy.

## Conclusions

When the number of genotyped animals exceeds the number of marker loci, the genomic relationship matrix cannot be full rank. We introduce an approach that partitions the genotyped animals into two sets, one of which can be referred to as core animals, and the other as non-core animals whose breeding values can be written as a linear function of the breeding values of core animals. The MME used for genomic prediction are then constructed with only the breeding values of the core animals, and with phenotpyes of the non-core animals contributing to the predictions for core animals through their linear relationships to the core animals. The estimated breeding values of the non-core animals are obtained as a linear function of the estimates of the breeding values of the core animals. This gives exact solutions for all animals. Another approach is to blend the genomic relationship matrix with a numerator relationship matrix or a scaled identity matrix to ensure the blended genomic relationship matrix is full rank. In that case, standard mixed model computing procedures can be used, but the increase in computing effort will be proportional to the cube of the number of animals genotyped. That effort can be reduced by approximating the inverse of the blended genomic relationship matrix using the Apy algorithm. That approximation also partitions the animals into core and non-core groups, but explicitly fits both sets of animals in the MME. In some cases, it has been reported that this approach gives useful approximations. However, the computing effort for that approximate approach is similar to that of the exact approach introduced here.

## Additional files

**Additional file 1**. Julia scripts and results. A Jupyter notebook showing Julia scripts and results for GBLUP by strategies I to IV and for Apy-GBLUP.

**Additional file 2**. Results when Apy propery does not hold. Julia notebook showing results by Apy approach when Apy property does not hold.

**Additional file 3**. Magnitude of non-diagonal elements of D with blended G. Julia notebook showing that matrix **D** of (15) has large non-diagonal elements relative to the diagonals.

## References

[1] Strandén I, Garrick DJ (2009). Technical note: derivation of equivalent computing algorithms for genomic predictions and reliabilities of animal merit. J Dairy Sci.

[2] Fernando RL. Genetic evaluation and selection using genotypic, phenotypic and pedigree information. In: Proceedings of the 6th World Congress on Genetics Applied to Livestock Production, Armidale, 11–16 January 1998, vol. 26. p. 329–36.

[3] Meuwissen THE, Hayes BJ, Goddard ME (2001). Prediction of total genetic value using genome-wide dense marker maps. Genetics.

[4] Nejati-Javaremi A, Smith C, Gibson JP (1997). Effect of total allelic relationship on accuracy of evaluation and response to selection. J Anim Sci.

[5] VanRaden PM (2008). Efficient methods to compute genomic predictions. J Dairy Sci.

[6] Harville DA (1976). Maximum likelihood approaches to variance component estimation and to related problems. J Am Stat Assoc.

[7] Henderson CR (1984). Applications of linear models in animal breeding.

[8] Legarra A, Ducrocq V (2012). Computational strategies for national integration of phenotypic, genomic, and pedigree data in a single-step best linear unbiased prediction. J Dairy Sci.

[9] Misztal I, Legarra A, Aguilar I (2014). Using recursion to compute the inverse of the genomic relationship matrix. J Dairy Sci.

[10] Misztal I (2015). Inexpensive computation of the inverse of the genomic relationship matrix in populations with small effective population size. Genetics.

[11] Fragomeni BO, Lourenco DAL, Tsuruta S, Masuda Y, Aguilar I, Legarra A, Lawlor TJ, Misztal I (2015). Hot topic: use of genomic recursions in single-step genomic best linear unbiased predictor (BLUP) with a large number of genotypes. J Dairy Sci.

[12] Lourenco DAL, Tsuruta S, Fragomeni BO, Masuda Y, Aguilar I, Legarra A, Bertrand JK, Amen TS, Wang L, Moser DW, Misztal I (2015). Genetic evaluation using single-step genomic best linear unbiased predictor in American Angus. J Anim Sci.

[13] Masuda Y, Misztal I, Tsuruta S, Legarra A, Aguilar I, Lourenco DAL, Fragomeni BO, Lawlor TJ (2016). Implementation of genomic recursions in single-step genomic best linear unbiased predictor for US Holsteins with a large number of genotyped animals. J Dairy Sci.

[14] Searle S (1966). Matrix algebra for the biological sciences.

[15] Misztal I, Legarra A, Aguilar I (2009). Computing procedures for genetic evaluation including phenotypic, full pedigree, and genomic information. J Dairy Sci.

[16] Aguilar I (2013). Genetic evaluation using unsymmetric single step genomic methodology with large number of genotypes. Interbull Bull.

[17] Janss L, de los Campos G, Sheehan N, Sorensen D (2012). Inferences from genomic models in stratified populations. Genetics.

[18] Ostersen T, Christensen OF, Madsen P, Henryon M (2016). Sparse single-step method for genomic evaluation in pigs. Genet Sel Evol.

[19] Golub GH, Van Loan CF (2012). Matrix computations.

[20] Quaas RL (1976). Computing the diagonal elements and inverse of a large numerator relationship matrix. Biometrics.

[21] Chang HL, Fernando RL, Grossman M (1991). On the principle underlying the tabular method to compute coancestry. Theor Appl Genet.

[22] Emik LO, Terrill CE (1949). Systematic procedures for calculating inbreeding coefficients. J Hered.

[23] Legarra A, Aguilar I, Misztal I (2009). A relationship matrix including full pedigree and genomic information. J Dairy Sci.

[24] Christensen OF, Lund MS (2010). Genomic prediction when some animals are not genotyped. Genet Sel Evol.

[25] Gianola D, de los Campos G, Hill WG, Manfredi E, Fernando R (2009). Additive genetic variability and the Bayesian alphabet. Genetics.

[26] Stranden I, Mantysaari EA. Comparison of some equivalent equations to solve single-step GBLUP. In: Proceedings of the 10th World Congress of Genetics Applied to Livestock Production, Vancouver, 17–22 August 2014.

[27] Pocrnic I, Lourenco DAL, Masuda Y, Legarra A, Misztal I (2016). The dimensionality of genomic information and its effect on genomic prediction. Genetics.

[28] Fernando RL, Dekkers JC, Garrick DJ (2014). A class of Bayesian methods to combine large numbers of genotyped and non-genotyped animals for whole-genome analyses. Genet Sel Evol.

