# Supplementary material for: An efficient exact method to obtain GBLUP and single-step GBLUP when the genomic relationship matrix is singular
Source: Genet Sel Evol. 2016 Oct 27;48:80. doi: 10.1186/s12711-016-0260-7 (PMC5082134; doi:10.1186/s12711-016-0260-7)
Supplement: Supplementary file 1 — Additional file 1. Julia scripts and results. A Jupyter notebook showing Julia scripts and results for GBLUP by strategies I to IV and for Apy-GBLUP. [file 12711_2016_260_MOESM1_ESM.pdf]

1.0.NumericalExample

June 8, 2016

Function to determine a set of linearly independent rows of SNP covariates

```
In [62]: function findCore(X)
             M = copy(float(X))
             n,p = size(M)
             rows = collect(1:n)
             cols = collect(1:p)
             for i=1:min(n,p)
                 selr = i:n
                 selc = i:p
                 v,indx  = findmax(abs(M[selr,selc]))
                 row,col = ind2sub(M[selr,selc],indx)
                 irow = i-1 + row
                 icol = i-1 + col
                 rows[i],rows[irow] = rows[irow],rows[i]
                 cols[i],cols[icol] = cols[icol],cols[i]
                 M[i,:],M[irow,:] = M[irow,:],M[i,:]
                 M[:,i],M[:,icol] = M[:,icol],M[:,i]
                 sel1 = (i+1):n
                 if abs(M[i,i]) < 1e-10
                     println("breaking at i = ", i)
                     return rows,cols,i-1,M
                 end
                 M[sel1,selc] -= (M[sel1,i]*M[i,selc])./M[i,i]
             end
             rows,cols,min(n,p),M
         end
```

```
Out[62]: findCore (generic function with 1 method)
```

Input SNP covariates

```
In [63]: snpDat = readdlm("snpDat.txt")
```

```
Out[63]: 7x4 Array{Float64,2}:
           0.0  0.0  -1.0  0.0
          -1.0  1.0   0.0  0.0
           1.0  0.0  -1.0  0.0
          -1.0  0.0   0.0  1.0
           0.0  1.0   0.0  1.0
           0.0  1.0  -1.0  0.0
           1.0  1.0  -1.0  0.0
```

Input pedigree, phenotype and breeding values

```
In [64]: data    = readdlm("pedDat.txt")

Out[64]: 7x5 Array{Float64,2}:
          1.0  0.0  0.0   99.25  -0.25
          2.0  0.0  0.0   97.92  -0.94
          3.0  0.0  0.0  103.2    1.12
          4.0  1.0  2.0   99.39  -1.01
          5.0  1.0  2.0  102.03   0.79
          6.0  1.0  3.0  100.59   0.18
          7.0  1.0  3.0  101.7    1.55

In [65]: pedDat = data[:,1:3]  # pedigree
         y      = data[:,4]    # phenotypes
         a      = data[:,5]    # breeding values
         nothing
```

Determine rank and set of linearly independent rows

```
In [66]: rows,cols,rank,RE = findCore(snpDat)

Out[66]: ([2,7,1,4,5,6,3],[1,2,3,4],4,
         7x4 Array{Float64,2}:
          -1.0  1.0   0.0  0.0
           0.0  2.0  -1.0  0.0
           0.0  0.0  -1.0  0.0
           0.0  0.0   0.0  1.0
           0.0  0.0   0.0  0.0
           0.0  0.0   0.0  0.0
           0.0  0.0   0.0  0.0)
```

The following rows are linearly independent:

```
In [67]: rows[1:rank]

Out[67]: 4-element Array{Int64,1}:
          2
          7
          1
          4
```

SNP covariates are now reordered such that the first 4 rows are linearly independent

```
In [68]: M = snpDat[rows,:]

Out[68]: 7x4 Array{Float64,2}:
          -1.0  1.0   0.0  0.0
           1.0  1.0  -1.0  0.0
           0.0  0.0  -1.0  0.0
          -1.0  0.0   0.0  1.0
           0.0  1.0   0.0  1.0
           0.0  1.0  -1.0  0.0
           1.0  0.0  -1.0  0.0
```

GBLUP by strategy I We are using $\sigma_e^2 = \sigma_u^2 = 1.0$ in the following calculations.

```
In [69]: Va   = 1.0
         Ve   = 1.0
         n,p = size(M)
         R   = eye(n)*Ve    # residual covariance matrix
         X   = ones(n,1)    # incidence matrix for μ
         G   = M*M'/p * Va  # genomic covariance matrix
```

```
Out[69]: 7x7 Array{Float64,2}:
           0.5    0.0   0.0    0.25  0.25  0.25  -0.25
           0.0    0.75  0.25  -0.25  0.25  0.5    0.5
           0.0    0.25  0.25   0.0   0.0   0.25   0.25
           0.25  -0.25  0.0    0.5   0.25  0.0   -0.25
           0.25   0.25  0.0    0.25  0.5   0.25   0.0
           0.25   0.5   0.25   0.0   0.25  0.5    0.25
          -0.25   0.5   0.25  -0.25  0.0   0.25   0.5
```

```
In [70]: V = G + R
```

```
Out[70]: 7x7 Array{Float64,2}:
           1.5    0.0   0.0    0.25  0.25  0.25  -0.25
           0.0    1.75  0.25  -0.25  0.25  0.5    0.5
           0.0    0.25  1.25   0.0   0.0   0.25   0.25
           0.25  -0.25  0.0    1.5   0.25  0.0   -0.25
           0.25   0.25  0.0    0.25  1.5   0.25   0.0
           0.25   0.5   0.25   0.0   0.25  1.5    0.25
          -0.25   0.5   0.25  -0.25  0.0   0.25   1.5
```

```
In [71]: Vi = inv(V)
         bHat = inv(X'Vi*X)*X'Vi*y[rows]           # GLS solution for μ, y has to be reordered
                                                   # in the same way as SNP covariates
```

```
Out[71]: 1-element Array{Float64,1}:
          100.432
```

```
In [72]: uHatS1 = zeros(n)
         uHatS1[rows] = G*Vi*(y[rows]-X*bHat)
         uHatS1                                   # GBLUP for animals ordered 1,2,...6,7
```

```
Out[72]: 7-element Array{Float64,1}:
           0.140752
          -0.94757
           1.08562
          -0.694411
           0.247757
           0.138051
           1.08292
```

GBLUP by strategy II

```
In [73]: lhs   = [X'X/Ve X'/Ve
                  G*X/Ve G /Ve + eye(7)]
         rhs   = [X'y[rows]/Ve
                  G*y[rows]/Ve]
```

```
sol          = lhs\rhs
uHatS2       = zeros(n)
println("GBLUP Results")
uHatS2[rows] = sol[2:end]
uHatS2
```

GBLUP Results

```
Out[73]: 7-element Array{Float64,1}:
           0.140752
          -0.94757
           1.08562
          -0.694411
           0.247757
           0.138051
           1.08292
```

Condition number for LHS of MME

```
In [74]: cond(lhs)

Out[74]: 10.91158791184995
```

GBLUP by strategy III

```
In [75]: λ    = Ve/Va
         M1   = M[1:rank,:]
         M2   = M[(rank+1):end,:]
         G11  = M1*M1'/p
         G21  = M2*M1'/p
         iG11 = inv(G11)
         LP   = G21*iG11
         W    = [eye(rank)
                 LP]
         lhs = [X'X X'W
             W'X W'W + iG11*λ]
         rhs = [X'y[rows]
                W'y[rows]]
         println("Solutions to p+r MME")
         sol  = lhs\rhs
```

Solutions to p+r MME

```
Out[75]: 5-element Array{Float64,1}:
          100.432
           -0.94757
            1.08292
            0.140752
           -0.694411
```

Condition number for LHS of MME

```
In [76]: cond(lhs)

Out[76]: 11.25924879396821
```

GBLUP Results

In [77]:
```
uHatS3       = zeros(n)
uHatS3[rows] = W*sol[2:end]                    # GBLUP for animals ordered 1,2,...6,7
uHatS3
```

Out[77]:
```
7-element Array{Float64,1}:
  0.140752
 -0.94757
  1.08562
 -0.694411
  0.247757
  0.138051
  1.08292
```

In [78]:
```
cor(uHatS3,a)
```

Out[78]:
```
0.9503330443623016
```

Apy-GBLUP

In [79]:
```
k     = 4
Mp   = M[1:k,:]    # core animals
My   = M[(k+1):end,:]
Gpp = Mp*Mp'/p

Gyp   = My*Mp'/p
iGpp = inv(Gpp)
P     = Gyp*iGpp
Gyy   = My*My'/p;
```

Note that when $k = 4$, $\mathbf{G}_{pp} = \mathbf{G}_{11}$, $\mathbf{G}_{yy} = \mathbf{G}_{22}$, $\mathbf{G}_{yp} = \mathbf{G}_{21}$, and $\mathbf{P} = \mathbf{L}'$

In [80]:
```
P
```

Out[80]:
```
3x4 Array{Float64,2}:
  0.0  1.0  -1.0  1.0
  0.5  0.5   0.5  0.0
 -0.5  0.5   0.5  0.0
```

Last 3 rows of G as a linear combination of the first 4 rows:

In [81]:
```
round(P*G[1:4,:],10)
```

Out[81]:
```
3x7 Array{Float64,2}:
  0.25  0.25  0.0    0.25  0.5   0.25  -0.0
  0.25  0.5   0.25   0.0   0.25  0.5    0.25
 -0.25  0.5   0.25  -0.25  0.0   0.25   0.5
```

Last 3 rows of G:

In [82]:
```
G[5:7,:]
```

Out[82]:
```
3x7 Array{Float64,2}:
  0.25  0.25  0.0    0.25  0.5   0.25  0.0
  0.25  0.5   0.25   0.0   0.25  0.5   0.25
 -0.25  0.5   0.25  -0.25  0.0   0.25  0.5
```

Last 3 columns of G as a linear combination of the first 4 columns:

```
In [83]: round(G[:,1:4]*P',10)

Out[83]: 7x3 Array{Float64,2}:
           0.25  0.25  -0.25
           0.25  0.5    0.5
           0.0   0.25   0.25
           0.25  0.0   -0.25
           0.5   0.25   0.0
           0.25  0.5    0.25
          -0.0   0.25   0.5
```

Last 3 columns of G:

```
In [84]: G[:,5:7]

Out[84]: 7x3 Array{Float64,2}:
          0.25  0.25  -0.25
          0.25  0.5    0.5
          0.0   0.25   0.25
          0.25  0.0   -0.25
          0.5   0.25   0.0
          0.25  0.5    0.25
          0.0   0.25   0.5
```

The matrix **D** can be written as:

$$\mathbf{D} = \mathbf{G}_{yy} - \mathbf{P}\mathbf{G}_{pp}\mathbf{P}'$$

or as

$$\mathbf{D} = \mathbf{G}_{yy} - \mathbf{G}_{yp}\mathbf{G}_{pp}^{-1}\mathbf{G}_{py}$$

```
In [85]: D = Gyy - P*Gpp*P'
         round(D,15)

Out[85]: 3x3 Array{Float64,2}:
          0.0  0.0  0.0
          0.0  0.0  0.0
          0.0  0.0  0.0
```

```
In [86]: D1 = Gyy - Gyp*iGpp*Gyp'
         round(D1,15)

Out[86]: 3x3 Array{Float64,2}:
          0.0  0.0  0.0
          0.0  0.0  0.0
          0.0  0.0  0.0
```

The Apy algorithm requies the inverse of **D**. So, if **D** is null, we set it to be a diagonal matrix with small values $s = 0001$. Whenever the Apy condition is met, **D** will be null.

```
In [87]: s = 0.0001
         d = diag(D)
         d1 = map(Float64,[i<1e-14 ? s:i for i in d])
         D = diagm(d1)
```

```
Out[87]: 3x3 Array{Float64,2}:
          0.0001  0.0     0.0
          0.0     0.0001  0.0
          0.0     0.0     0.0001
```

```
In [88]: T = [-P'
             eye(Gyy)]
         iGApy = [iGpp        zeros(Gyp')
                  zeros(Gyp)  zeros(Gyy)] + T*inv(D)*T'
```

```
Out[88]: 7x7 Array{Float64,2}:
           5003.0      -1.0       1.0      -2.0       0.0  -5000.0   5000.0
             -1.0   15003.0   -5003.0   10002.0  -10000.0  -5000.0  -5000.0
              1.0   -5003.0   15007.0  -10002.0   10000.0  -5000.0  -5000.0
             -2.0   10002.0  -10002.0   10004.0  -10000.0      0.0      0.0
              0.0  -10000.0   10000.0  -10000.0   10000.0      0.0      0.0
          -5000.0   -5000.0   -5000.0       0.0       0.0  10000.0      0.0
           5000.0   -5000.0   -5000.0       0.0       0.0      0.0  10000.0
```

Note that in this inverse, the submatrix corresponding to $\mathbf{G}_{yy}$ is diagonal. Direct inversion of $\mathbf{G}^*$ obtained by adding s to the diagonals of $\mathbf{G}$ corresponding to the animals in the young group gives the same result:

```
In [89]: round(inv(G + diagm([0;0;0;0;s;s;s])),4)
```

```
Out[89]: 7x7 Array{Float64,2}:
           5003.0      -1.0       1.0      -2.0       0.0  -5000.0   5000.0
             -1.0   15003.0   -5003.0   10002.0  -10000.0  -5000.0  -5000.0
              1.0   -5003.0   15007.0  -10002.0   10000.0  -5000.0  -5000.0
             -2.0   10002.0  -10002.0   10004.0  -10000.0      0.0      0.0
             -0.0  -10000.0   10000.0  -10000.0   10000.0      0.0     -0.0
          -5000.0   -5000.0   -5000.0       0.0      -0.0  10000.0     -0.0
           5000.0   -5000.0   -5000.0       0.0      -0.0     -0.0  10000.0
```

But, adding s to all the diagonals will not result in a sparse inverse:

```
In [90]: round(inv(G + diagm([s;s;s;s;s;s;s])),4)
```

```
Out[90]: 7x7 Array{Float64,2}:
           3334.67       0.0          0.0      ...      -0.6664  -3332.67     3332.67
             -0.0      5000.5        -0.4998     -2499.5     -2500.0     -2500.0
              0.0        -0.4998   5001.5         2499.0     -2499.5     -2499.5
             -0.6664   2499.5     -2499.0        -2499.75       -0.0833      0.5831
             -0.6664  -2499.5      2499.0         2501.75       -0.5831      0.0833
          -3332.67    -2500.0     -2499.5      ...      -0.5831   5833.92     -833.417
           3332.67    -2500.0     -2499.5              0.0833   -833.417    5833.92
```

```
In [91]: Z = eye(n)
         lhs = [X'X X'Z
                Z'X Z'Z + iGApy*λ]
         rhs = [X'y[rows]
                Z'y[rows]]
         sol = lhs\rhs
         uHatApy = zeros(n)
         uHatApy[rows] = sol[2:end]
         cor(uHatApy,a)
```

```
Out[91]: 0.9503383921115987
```

Condition number for LHS of MME

```
In [92]: cond(lhs)
```

```
Out[92]: 56457.64045097132
```

Apy-GBLUP with Blending

```
In [93]: using PedModule
         ped=PedModule.mkPed("ped.txt")
         ped.idMap
         PedModule.getIDs(ped)          # Access the reordered IDs
         Ainv=PedModule.AInverse(ped)
         AA = inv(full(Ainv))
         indx = [ped.idMap[dec(i)].seqID for  i in 1:7]
         A = AA[indx,indx]
         G = 0.95*M*M'/p + 0.05*A[rows,rows]
         k = 4
         Sc = 1:k
         Sn = (k+1):7
         Gpp = G[Sc,Sc]
         Gyp = G[Sn,Sc]
         iGpp = inv(Gpp)
         P    = Gyp*iGpp
         Gyy  = G[Sn,Sn]
         D = Gyy - P*Gpp*P'
         s = 0.0001
         d = diag(D)
         d1 = map(Float64,[i<1e-14 ? s:i for i in d])
         D = diagm(d1)
         T = [-P'
             eye(Gyy)]
         iGApy = [iGpp        zeros(Gyp')
             zeros(Gyp)  zeros(Gyy)] + T*inv(D)*T'
         Z = eye(n)
         λ    = Ve/Va
         lhs = [X'X X'Z
                Z'X Z'Z + iGApy*λ]
         rhs = [X'y[rows]
                Z'y[rows]]
         sol = lhs\rhs
         uHatApyBlend = zeros(n)
         uHatApyBlend[rows] = sol[2:end]
         cor(uHatApyBlend,a)
```

```
Out[93]: 0.951312416613279
```

Condition number for LHS of MME

```
In [94]: cond(lhs)
```

```
Out[94]: 62.053857285657045
```

GBLUP by strategy IV

In [95]:
```
k   = size(M,2)
λ   = Ve/Va*k
U,R = qr(M')
p   = size(U,1)
Z   = R'
lhs = [X'X X'Z
       Z'X Z'Z + eye(p)*λ]
rhs = [X'y[rows]
       Z'y[rows] ]
sol  = lhs\rhs
uHatS4 = zeros(n)
uHatS4[rows] = Z*sol[2:end]
rEXT = cor(uHatS4,a)
```

Out[95]: 0.9503330443623

In [96]:
```
cond(lhs)
```

Out[96]: 6.761107168073085

0.0.1 GBLUP results from strategies I through IV, and from Apy-GBLUP with and without blending

True breeding values are in the last column

In [97]:
```
[uHatS1 uHatS2 uHatS3 uHatS4 uHatApyBlend uHatApy a]
```

Out[97]: 7x7 Array{Float64,2}:

0.140752	0.140752	0.140752	0.140752	0.101705	0.14076	-0.25
-0.94757	-0.94757	-0.94757	-0.94757	-0.944249	-0.947539	-0.94
1.08562	1.08562	1.08562	1.08562	1.13924	1.08577	1.12
-0.694411	-0.694411	-0.694411	-0.694411	-0.704425	-0.694399	-1.01
0.247757	0.247757	0.247757	0.247757	0.259407	0.247887	0.79
0.138051	0.138051	0.138051	0.138051	0.141521	0.138067	0.18
1.08292	1.08292	1.08292	1.08292	1.05594	1.08291	1.55

0.0.2 GBLUP by strategy I for blended G

In [105]:
```
Va  = 1.0
Ve  = 1.0
n,p = size(M)
R   = eye(n)*Ve     # residual covariance matrix
X   = ones(n,1)     # incidence matrix for μ
G   = M*M'/p * Va   # genomic covariance matrix
G   = 0.95G + 0.05A[rows,rows]
V = G + R
Vi = inv(V)
bHat = inv(X'Vi*X)*X'Vi*y[rows]           # GLS solution for μ, y has to be reordered
uHatS1Blend = zeros(n)
uHatS1Blend[rows] = G*Vi*(y[rows]-X*bHat)
cor(uHatS1Blend,a)
```

Out[105]: 0.9497630140427739

0.0.3 GBLUP results for blended G from strategy I and Apy-GBLUP

In [106]:
```
[uHatS1Blend  uHatApyBlend]
```

Out[106]:
```
7x2 Array{Float64,2}:
  0.0977627   0.101705
 -0.950329   -0.944249
  1.11345     1.13924
 -0.705481   -0.704425
  0.22868     0.259407
  0.155438    0.141521
  1.05505     1.05594
```

0.0.4 Application of Apy to invert A

In [100]:
```
k    = 4
AA   = A[rows,rows] # reordered
App  = AA[1:4,1:4]
Ayp  = AA[5:7,1:4]
iApp = inv(App)
P    = Ayp*iApp
Ayy  = AA[5:7,5:7]
D = Ayy - P*App*P'
round(D,5)
```

Out[100]:
```
3x3 Array{Float64,2}:
 0.5  0.0      0.0
 0.0  0.66667  0.33333
 0.0  0.33333  0.66667
```

Apy uses only the diagonals from D

In [101]:
```
d = diag(D)
D = diagm(d)
```

Out[101]:
```
3x3 Array{Float64,2}:
 0.5  0.0       0.0
 0.0  0.666667  0.0
 0.0  0.0       0.666667
```

Inverse of A from Apy

In [102]:
```
T = [-P'
    eye(Ayy)]
iApy = [iApp        zeros(Ayp')
        zeros(Ayp)  zeros(Ayy)] + T*inv(D)*T'
round(iApy,3)
```

Out[102]:
```
7x7 Array{Float64,2}:
  2.0  -0.0     1.0    -1.0  -1.0   0.0   0.0
 -0.0   2.167  -0.833   0.0   0.0  -0.5  -1.0
  1.0  -0.833   2.667  -1.0  -1.0  -0.5   0.5
 -1.0   0.0    -1.0     2.0   0.0   0.0  -0.0
 -1.0   0.0    -1.0     0.0   2.0   0.0   0.0
  0.0  -0.5    -0.5     0.0   0.0   1.5   0.0
  0.0  -1.0     0.5    -0.0   0.0   0.0   1.5
```

Exact inverse of A

```
In [103]: round(inv(AA),3)

Out[103]: 7x7 Array{Float64,2}:
            2.0  -0.0   1.0  -1.0  -1.0  -0.0   0.0
           -0.0   2.0  -1.0   0.0   0.0   0.0  -1.0
            1.0  -1.0   3.0  -1.0  -1.0  -1.0   1.0
           -1.0   0.0  -1.0   2.0   0.0   0.0  -0.0
           -1.0   0.0  -1.0   0.0   2.0   0.0  -0.0
           -0.0   0.0  -1.0   0.0   0.0   2.0  -1.0
            0.0  -1.0   1.0  -0.0  -0.0  -1.0   2.0
```

Difference between exact and Apy inverse matrices

```
In [104]: round(iApy-inv(AA),3)

Out[104]: 7x7 Array{Float64,2}:
            0.0  -0.0    -0.0     0.0   0.0   0.0   0.0
            0.0   0.167   0.167  -0.0  -0.0  -0.5   0.0
           -0.0   0.167  -0.333   0.0   0.0   0.5  -0.5
            0.0   0.0     0.0    -0.0  -0.0  -0.0  -0.0
            0.0  -0.0     0.0    -0.0   0.0  -0.0   0.0
            0.0  -0.5     0.5    -0.0  -0.0  -0.5   1.0
           -0.0   0.0    -0.5     0.0   0.0   1.0  -0.5
```
