# Supplementary material for: An efficient exact method to obtain GBLUP and single-step GBLUP when the genomic relationship matrix is singular
Source: Genet Sel Evol. 2016 Oct 27;48:80. doi: 10.1186/s12711-016-0260-7 (PMC5082134; doi:10.1186/s12711-016-0260-7)
Supplement: Supplementary file 2 — Additional file 2. Results when Apy propery does not hold. Julia notebook showing results by Apy approach when Apy property does not hold. [file 12711_2016_260_MOESM2_ESM.pdf]

1.1.NumericalExample

February 28, 2016

Apy-GBLUP

```
In [167]: k    = 3
          Mp   = M[1:k,:]    # core animals
          My   = M[(k+1):end,:]
          Gpp  = Mp*Mp'/p

          Gyp  = My*Mp'/p
          iGpp = inv(Gpp)
          P    = Gyp*iGpp
          Gyy  = My*My'/p;
```

```
In [168]: P
```

```
Out[168]: 4x3 Array{Float64,2}:
            0.5  -0.5   0.5
            0.5   0.5  -0.5
            0.5   0.5   0.5
           -0.5   0.5   0.5
```

```
In [169]: round(P*G[1:k,:],10)
```

```
Out[169]: 4x7 Array{Float64,2}:
            0.25  -0.25  -0.0    0.25  0.0   0.0   -0.25
            0.25   0.25   0.0    0.0   0.25  0.25   0.0
            0.25   0.5    0.25   0.0   0.25  0.5    0.25
           -0.25   0.5    0.25  -0.25  0.0   0.25   0.5
```

```
In [170]: G[(k+1):end,:]
```

```
Out[170]: 4x7 Array{Float64,2}:
            0.25  -0.25  0.0    0.5   0.25  0.0   -0.25
            0.25   0.25  0.0    0.25  0.5   0.25   0.0
            0.25   0.5   0.25   0.0   0.25  0.5    0.25
           -0.25   0.5   0.25  -0.25  0.0   0.25   0.5
```

```
In [171]: D = Gyy - P*Gpp*P'
          round(D,15)
```

```
Out[171]: 4x4 Array{Float64,2}:
           0.25  0.25  -0.0  0.0
           0.25  0.25   0.0  0.0
           0.0   0.0    0.0  0.0
           0.0   0.0    0.0  0.0
```

The Apy algorithm requies the inverse of $\mathbf{D}$. So, diagonal elements that are null are replaced with small value $s = 0001$.

```
In [174]: s = 0.0001
          d = diag(D)
          d1 = map(Float64,[i<1e-14 ? s:i for i in d])
          D = diagm(d1)

Out[174]: 4x4 Array{Float64,2}:
           0.25  0.0   0.0     0.0
           0.0   0.25  0.0     0.0
           0.0   0.0   0.0001  0.0
           0.0   0.0   0.0     0.0001

In [175]: T = [-P'
              eye(Gyy)]
          iGApy = [iGpp        zeros(Gyp')
                   zeros(Gyp)  zeros(Gyy)] + T*inv(D)*T'

Out[175]: 7x7 Array{Float64,2}:
            5004.0      0.0      0.0  -2.0  -2.0  -5000.0   5000.0
               0.0   5004.0   4996.0   2.0  -2.0  -5000.0  -5000.0
               0.0   4996.0   5008.0  -2.0   2.0  -5000.0  -5000.0
              -2.0      2.0     -2.0   4.0   0.0      0.0      0.0
              -2.0     -2.0      2.0   0.0   4.0      0.0      0.0
           -5000.0  -5000.0  -5000.0   0.0   0.0  10000.0      0.0
            5000.0  -5000.0  -5000.0   0.0   0.0      0.0  10000.0

In [181]: Z = eye(n)
          lhs = [X'X X'Z
                 Z'X Z'Z + iGApy*λ]
          rhs = [X'y[rows]
                 Z'y[rows]]
          sol = lhs\rhs
          uHatApy = zeros(n)
          uHatApy[rows] = sol[2:end];
```

0.0.1 GBLUP results from strategies I, II and III, and from Apy-GBLUP(k=3)

True breeding values are in the last column

```
In [182]: [uHatS1 uHatS2 uHatS3 uHatApy a]

Out[182]: 7x5 Array{Float64,2}:
            0.140752   0.140752   0.140752   0.128189   -0.25
           -0.94757   -0.94757   -0.94757   -0.948948   -0.94
            1.08562    1.08562    1.08562    1.04031     1.12
           -0.694411  -0.694411  -0.694411  -0.949783   -1.01
            0.247757   0.247757   0.247757   0.278184    0.79
            0.138051   0.138051   0.138051   0.0911944   0.18
            1.08292    1.08292    1.08292    1.00315     1.55
```

The matrix $\mathbf{G}^*$ that is inverted in the Apy algorithm now deviates subtantially from $\mathbf{G}$.

```
In [179]: round(inv(iGApy) - G, 10)
```

```
Out[179]: 7x7 Array{Float64,2}:
 -0.0  0.0  0.0  -0.0   -0.0   0.0     0.0
 -0.0  0.0  0.0  -0.0   -0.0   0.0     0.0
 -0.0  0.0  0.0  -0.0   -0.0   0.0     0.0
 -0.0  0.0  0.0  -0.0   -0.25  0.0     0.0
 -0.0  0.0  0.0  -0.25  -0.0   0.0     0.0
 -0.0  0.0  0.0  -0.0   -0.0   0.0001  0.0
 -0.0  0.0  0.0  -0.0   -0.0   0.0     0.0001
```
