# Supplementary material for: An efficient exact method to obtain GBLUP and single-step GBLUP when the genomic relationship matrix is singular
Source: Genet Sel Evol. 2016 Oct 27;48:80. doi: 10.1186/s12711-016-0260-7 (PMC5082134; doi:10.1186/s12711-016-0260-7)
Supplement: Supplementary file 3 — Additional file 3. Magnitude of non-diagonal elements of D with blended G. Julia notebook showing that matrix D of (15) has large non-diagonal elements relative to the diagonals. [file 12711_2016_260_MOESM3_ESM.pdf]

Additional File 3

October 6, 2016

0.1 Magnitude of Non-diagonal Elements of D With Blended G

Function to determine a set of linearly independent rows of SNP covariates Animals with these genotypes will be used as the core.

```
In [2]: function findCore(X)
            M = copy(float(X))
            n,p = size(M)
            rows = collect(1:n)
            cols = collect(1:p)
            for i=1:min(n,p)
                selr = i:n
                selc = i:p
                v,indx  = findmax(abs(M[selr,selc]))
                row,col = ind2sub(M[selr,selc],indx)
                irow = i-1 + row
                icol = i-1 + col
                rows[i],rows[irow] = rows[irow],rows[i]
                cols[i],cols[icol] = cols[icol],cols[i]
                M[i,:],M[irow,:] = M[irow,:],M[i,:]
                M[:,i],M[:,icol] = M[:,icol],M[:,i]
                sel1 = (i+1):n
                if abs(M[i,i]) < 0.0000000001
                    println("breaking at i = ", i)
                    return rows,cols,i-1,M
                end
                M[sel1,selc] -= (M[sel1,i]*M[i,selc])./M[i,i]
            end
            rows,cols,min(n,p),M
        end
```

```
Out[2]: findCore (generic function with 1 method)
```

Input SNP covariates

```
In [12]: snpDat = readdlm("snpDat.txt")
         p = size(snpDat,2);
```

Input pedigree, phenotype and breeding values

```
In [4]: data   = readdlm("pedDat.txt")
```

```
Out[4]: 7x5 Array{Float64,2}:
         1.0  0.0  0.0   99.25  -0.25
         2.0  0.0  0.0   97.92  -0.94
```

```
 3.0  0.0  0.0  103.2    1.12
 4.0  1.0  2.0   99.39  -1.01
 5.0  1.0  2.0  102.03   0.79
 6.0  1.0  3.0  100.59   0.18
 7.0  1.0  3.0  101.7    1.55
```

```
In [5]: pedDat = data[:,1:3]  # pedigree
        y      = data[:,4]    # phenotypes
        a      = data[:,5]    # breeding values
        nothing
```

Determine rank and set of linearly independent rows

```
In [6]: rows,cols,rank,RE = findCore(snpDat)
```

```
Out[6]: ([2,7,1,4,5,6,3],[1,2,3,4],4,
        7x4 Array{Float64,2}:
         -1.0  1.0   0.0  0.0
          0.0  2.0  -1.0  0.0
          0.0  0.0  -1.0  0.0
          0.0  0.0   0.0  1.0
          0.0  0.0   0.0  0.0
          0.0  0.0   0.0  0.0
          0.0  0.0   0.0  0.0)
```

The following rows are linearly independent:

```
In [7]: rows[1:rank]
```

```
Out[7]: 4-element Array{Int64,1}:
         2
         7
         1
         4
```

SNP covariates are now reordered such that the first 4 rows are linearly independent

```
In [8]: M = snpDat[rows,:]
```

```
Out[8]: 7x4 Array{Float64,2}:
         -1.0  1.0   0.0  0.0
          1.0  1.0  -1.0  0.0
          0.0  0.0  -1.0  0.0
         -1.0  0.0   0.0  1.0
          0.0  1.0   0.0  1.0
          0.0  1.0  -1.0  0.0
          1.0  0.0  -1.0  0.0
```

Apy Calculations

```
In [15]: using JWAS.PedModule
         ped  = PedModule.mkPed("ped.txt")
         Ainv = PedModule.AInverse(ped)
         AA   = inv(full(Ainv))
         indx = [ped.idMap[dec(i)].seqID for  i in 1:7]
         A    = AA[indx,indx]
```

```
G    = 0.95*M*M'/p + 0.05*A[rows,rows]
k    = 4
Sc   = 1:k
Sn   = (k+1):7
Gcc  = G[Sc,Sc]
Gnc  = G[Sn,Sc]
iGcc = inv(Gcc)
P    = Gnc*iGcc
Gnn  = G[Sn,Sn]
P;
```

```
Finished!
```

The matrix **D** can be written as:

$$\mathbf{D} = \mathbf{G}_{nn} - \mathbf{P}\mathbf{G}_{cc}\mathbf{P}'$$

or as

$$\mathbf{D} = \mathbf{G}_{nn} - \mathbf{G}_{nc}\mathbf{G}_{nn}^{-1}\mathbf{G}_{cn}$$

In [16]:
```
D = Gnn - P*Gcc*P'
round(D,15)
```

Out[16]:
```
3x3 Array{Float64,2}:
  0.0883634    0.000213913  -0.0204569
  0.000213913  0.0481462     0.0119978
 -0.0204569    0.0119978     0.0679162
```

Note that **D** is not diagonal and has values that are large relative to the diagonals. As the dimension of D gets larger, the consequence of these non-diagonals will be greater.
